# Oncolytic virotherapy evolved into the fourth generation as tumor immunotherapy

**DOI:** 10.1186/s12967-023-04360-8

**Published:** 2023-07-25

**Authors:** Xianwang Wang, Yihua Shen, Xingxia Wan, Xiaoqing Hu, Wen-Qi Cai, Zijun Wu, Qiang Xin, Xiaoqing Liu, Jingang Gui, Hong-Yi Xin, Hong-Wu Xin

**Affiliations:** 1 Department of Biochemistry and Molecular Biology, Health Science Center, Yangtze University, Jingzhou, 434023 Hubei China; 2 College of Arts and Sciences, Yangtze University, Jingzhou, 434023 Hubei China; 3 The Second School of Clinical Medicine, Yangtze University, Jingzhou, 434023 Hubei China; 4 Xinzhou Traditional Chinese Medicine Hospital, Zhongnan Hospital of Wuhan University (Xinzhou), Wuhan, 430000 Hubei China; 5 School of Graduate Students, Inner Mongolia Medical University, Inner Mongolian Autonomous Region, Hohhot, 010110 China; 6 Laboratory of Tumor Immunology, Beijing Pediatric Research Institute, Beijing Children’s Hospital, Capital Medical University, National Center for Children’s Health, Beijing, 100045 China; 7 The Doctoral Scientific Research Center, People’s Hospital of Lianjiang, Guangdong, 524400 China; 8 The Doctoral Scientific Research Center, Affiliated People’s Hospital of Lianjiang, Guangdong Medical University, Guangdong, 524400 China

**Keywords:** Oncolytic virotherapy (OVT), Oncolytic viruses (OVs), Cancer, T-VEC, BiTA

## Abstract

**Background:**

Oncolytic virotherapy (OVT) is a promising anti-tumor modality that utilizes oncolytic viruses (OVs) to preferentially attack cancers rather than normal tissues. With the understanding particularly in the characteristics of viruses and tumor cells, numerous innovative OVs have been engineered to conquer cancers, such as Talimogene Laherparepvec (T-VEC) and tasadenoturev (DNX-2401). However, the therapeutic safety and efficacy must be further optimized and balanced to ensure the superior safe and efficient OVT in clinics, and reasonable combination therapy strategies are also important challenges worthy to be explored.

**Main body:**

Here we provided a critical review of the development history and status of OVT, emphasizing the mechanisms of enhancing both safety and efficacy. We propose that oncolytic virotherapy has evolved into the fourth generation as tumor immunotherapy. Particularly, to arouse T cells by designing OVs expressing bi-specific T cell activator (BiTA) is a promising strategy of killing two birds with one stone. Amazing combination of therapeutic strategies of OVs and immune cells confers immense potential for managing cancers. Moreover, the attractive preclinical OVT addressed recently, and the OVT in clinical trials were systematically reviewed.

**Conclusion:**

OVs, which are advancing into clinical trials, are being envisioned as the frontier clinical anti-tumor agents coming soon.

## Introduction

Cancer is still a serious threat to human health and a major cause of death worldwide, even among adolescents and young adults [1, 2]. The scientists have been pursuing the ideal tumor prevention and treatment strategies all the time. Numerous promising tactics have been well developed, such as immunotherapy, photodynamic therapy and oncolytic virotherapy (OVT) [3–6].

OVT has its unique advantages and prospects, because oncolytic viruses (OVs) preferentially infect and replicate in tumor cells and destroy them, while leaving healthy cells largely untouched [7]. With increasingly high therapeutic efficacy being achieved recent years and owing to the unique features such as specific tumor tropism, low cytotoxicity against normal cells, OVT has been inviting a great attention as an ideal weapon against cancers.

**Fig. 1:**
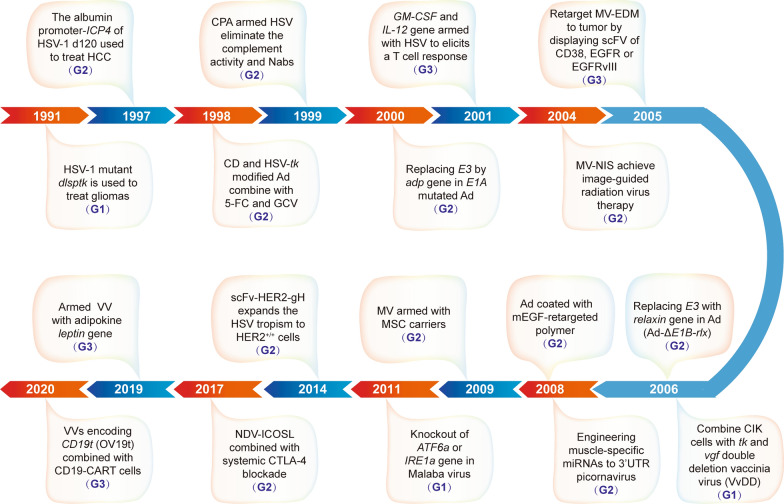
A timeline of milestones in the evolution of OVT

OVT has a long development history. Originally, viruses were known as the cause of human diseases, including some cancers [8]. It was not until early 1950s that the potential of viruses as anti-cancer agents had been recognized and applied [7, 9]. At that time, the application of tumor treatment with the spontaneous viruses or wild type viruses which quite often being scavenged by immune system, merely induces a subtle inhibition to tumor progression in patients. Meanwhile, these non-engineered viruses sometimes inevitably infect and spread to normal tissues, indiscriminately killed both tumor and normal cells, causing a series of unpredicted side effects. Therefore, safety and efficacy were the greatest challenges for the development of OVT. With the leap of gene cloning in the molecular virology, the scientists focus on improvement of their antitumor specificity and efficiency by manipulating the viral genomes. As shown in Fig. 1, we propose that OVT can be divided into the following four phases of development. The viruses originally used for treatment are usually spontaneous viruses. The first generation (G1) of engineered OVs mainly focus on manipulating within virus genome. By the genetic recombination the viruses were conferred with high specificity against tumor cells without targeting normal tissues. The first application of virotherapy with the engineered thymidine kinase (TK)-deficient herpes simplex viruses (HSV) was initiated in 1991 [10]. The second generation (G2) of engineered OVs armed with viral and/or non-viral genes. A series of chimeric viruses strategies, such as transductional targeting, transcriptional targeting, micro-RNA targeting and DNA shuffling approaches have been developed for restricting virus infection and toxicity in off-target tissues [11–13]. For example, Myb34.5, a second-generation replication-conditional HSV-1, has been exploited to target and dampen the pancreatic tumors [13]. Moreover, HSV engineered in gH of a scFv targeting the cancer-specific HER2 receptor, scFv-HER2-gH chimera, can enter, replicate and kill cancer cells efficiently [14]. The  third generation (G3) OVs were engineered with multiple coordinated viral and non-viral genes for tumor immunotherapy. Rivadeneira et al. demonstrates that intratumoral delivery of leptin by a VV can metabolically enhance tumor-infiltrating lymphocytes (TILs) effector and memory functions through improved mitochondrial oxidative phosphorylation, thereby potentiating therapeutic efficacy [15]. Anthony et al. engineered the vaccinia virus to express a nonsignaling, truncated CD19 (CD19t) protein for tumor-selective delivery, enabling targeting by CD19-CAR T cells [16]. Keeping stringency on tumor specificity and normal tissues safety usually hampers replicative fitness of viruses in target tissues. Thus, scientists keep pursuing ideal OVs that are highly tumor-specific without an attenuated clinical efficacy. In the first place, OVs have been designed to eliminate infected cancer cells by taking advantage of some of the most important properties of viruses or immune responses, including direct oncolysis, antitumor immunity, vascular-disrupting effects and bystander killing effect [17]. Secondly, along with the improvement of the tumor specificity, scientists are also constantly boosting the potency of OVT via prodrug activation, radiosensitization, immunostimulation and so on [18–23]. Worth to be noted, among these designs a second-generation oncolytic HSVs expressing TNF-α are being developed for cancer therapy and exerting its high efficacy for cancer therapy [24].

Until now, more than twenty different virus families have been engineered for cancer therapy, including but not limited to HSV, adenovirus (Ad), measles virus (MV), Newcastle disease virus (NDV), vaccinia virus (VV), reovirus, myxomavirus, poliovirus, poxviruses, vesicular stomatitis virus (VSV) [7, 19, 25–27]. These engineered viruses usually focused on targeting replication of OVs in the tumor bed, initiation of an immune-stimulating or immune-recruiting inflammatory response and exposure of tumor-associated antigens that can be targeted by the immune system [28]. Moreover, the safety and efficacy of OVs in combination with other treatments have been explored continuously [28–30]. Arming strategies that combine chemo-, radio- and immuno-therapies with OVT will be strengthened by greater viruses replication and spread [19, 30–36]. In this review, the summary of the knowledge on the OVT, including the development history, the applications of preclinical studies, the mechanism of enhancing the safety and efficacy, and clinical trials were provided. In addition, the most important attractive schemes of genetic modifications and combinatorial regimens with OVs were highlighted.

## OVs in preclinical development

As a promising cancer therapy strategy, OVT has immeasurable application potential, bringing a bright future to cancer patients. Many natural and genetically engineered OVs have been developed and underwent pre-clinical research stages (Table 1). Although the idea of using viruses to treat cancer originated in 1950s and has been around for more than 70 years, the modern era of OVT can be traced back to a 1991 cornerstone study, in which a *TK* gene was deleted in HSV with attenuated neurovirulence was shown to be active in a murine glioblastoma model [10]. Subsequently, the OVT upsurged globally and made great advance. The researchers began immersing themselves in manipulating various modifications with different types of viruses and testing them in animal models.

**Table 1:** The preclinical studies of OVs

Virus name, type and strain	Virus short name	Viral gene modification	Non-viral gene addition	Cell culture, mouse, human	Tumor type	Virus administration route, dose, times	Combination therapy	Safety (Major AEs)	Efficacy, (Tumor size, Survival benefit)	Novelty, advantages	Comments, disadvantages	Refs
HSV-1	oHSV-1			6 to 8-week-old NOD. Cg-*Prkdc*^*scid*^* Il2rg*^*tm1Wjl*^/SzJ (NSG) mice	Breast cancer, brain cancer, BCBMs	Intracranially infused with EGFR-CAR NK-92, oHSV-1, NK-92-EV	EGFR-CAR NK-92 cells		Suppression of tumor growth and significantly longer survival	Optimal efficacy in patients with EGFR + tumor		[36]
HSV-1	UV-HSV-1			NRG-3GS mice (15 weeks)	leukemia	3 × 10^6^ human PBMCs + 0.1 pfu/PBMC for 16 h	IL-15		Prolonged survival of T cell-depleted PBMCs mice	*Herpesviridae* members are potent stimulators of innate immune function	Future allogeneic mononuclear cell or NK infusion	[37]
HSV-1	oHSV-1			Female athymic nu/nu mice	GBM	IP, with PBS/ bortezomib (0.8 mg/kg) twice a week	Bortezomib		Necrosis in tumors	NK cell adjuvant therapy, virotherapy and proteasome blockade	Toxicities may be discovered	[155]
HSV	oHSV, MG18L			PARP*i*-sensitive or -resistant GSC. Female athymic mice	GBM	IP, olaparib (50 mg/kg) or vehicle; IT, MG18L or PBS	PARP*i,* Olaparib		Greatly extended survival	Applicable not only to GBM, but also to other tumor types	Treatment schedule not optimized	[33]
HSV	MG18L			7–8-week-old female SCID mice	GBM	Galunisertib (100 mg/kg), oral gavage daily from day 7 to 16. IT, MG18L (1 × 10^6^ pfu/3 µl) on day 9	TβR inhibitors SB431542, galunisertib		Cures in 60% of mice bearing orthotopic recurrent GBM	A novel synergistic interaction of oHSV therapy and TGF-β signaling blockade	Effect for initiating poorly invasiveGBM	[156]
HSV-1	oHSV			Mouse model of ATC	PDTC ATC	A single injection into the tumor using a Hamilton syringe	40 mg/kg of BRAFi (PLX4720) by oral gavage daily		Tumor reduced by 50% and inflammatory	Activated NK and T cells, and successfully incorporated anti-CTLA-4 or anti-PD-1		[157]
HSV	oHSV	γ1-34.5 deleted		6- 8-week-old C57BL/6 mice	MPNSTs	C134 (3.5 × 107 in 100 μL 10% glycerol in PBS) IT on day 4 and a week later	3 doses of RUX (INCB018424, AbexBio; 60 mg/kg) daily IP		Antitumor antigen and an antiviral responses	CD8 + T cell activation indispensable for the antitumor benefit	CTL response not been fully investigated	[31]
HSV	oHSV		Vstat120, anti-angiogenic	Female BALB/C mice or Bai1 wildtype or knockout C57/Bl/6 mice (littermates)	GBM	IT, HBSS/PBS, rHSVQ1, or RAMBO virus (1 × 10^5^ PFU/mouse)		Transient weight loss	Reduced macrophages/microglia, increased virus replication	Shielded from inflammatory macrophage antiviral response, without reducing safety	How Vstat12 blocks BAI1 unclear	[39]
HSV	oHSV-TRAIL		TRAIL	Athymic mice (6 weeks of age); TMZ-resistant primary and recurrent GSC	GBM	IT, 3–6 μl, 2.0 × 10^6^ pfu, twice on days 14 and 26			Prolong survival through robust apoptosis	Potent therapeutic efficacy		[158]
HSV	MSC-oHSV		MSCs	a BRAF mutant line from BrafV600E/wtCdkn2A^−/−^Pten^−/−^ mice	Melanoma	ICA, intracarotid injection	PD-L1 blockade		Significantly prolongs the survival	Target melanoma brain metastasis		[72]
HSV-1	oHSV-1- SU4-124	ICP4 under survivin promoter	Rat FGF2 5’UTR in front of ICP4 ORF	Female C57BL6 mice	Glioma U87	IT, 100 mm3 tumor, 3galΔ3, or CMV-ICP4 HSV-1 or SU4-124 HSV-1			a significantly enhanced antitumor effect	Triple-regulated ICP4 gene expressed from an amplicon to supplement a replication-defective HSV-1		[38]
HSV	oHSV- G47Δ	G47Δ-mCherry, G47Δ-Us11-fluc	G47Δ	MN3 cells, 7–8-week-old female SCID mice	Meningiomas	IT, 2 G47Δ injections (2 × 10^6^ pfu/3 µL)			Significantly prolonged survival	Efficacy against several patient-derived meningioma lines of different grade	To study MN3 as CSC	[159]
HSV	oHSVG47Δ(G47Δ-mIL12)	G47Δ	IL-12	Female C57Bl/6 mice (8–9 weeks)	GBM	IT, G47Δ-mIL12 in 2 μl	Anti-CTLA-4, anti-PD-1		89% long-term survivors; the cure rate 4/6 and 5/7	Synergistic effect and inducing immunological memory	Lack of representative murine models	[32]
Ad	oAd- CARsc-pSia		Bispecific adapter CARsc-pSia	C57BL/6 and NMRI-nu/nu mice	SCLC	IV pretreated with CARsc-pSia (15 μg/250 μL/mouse) or PBS	hTERT-AdLuc (1 × 10^9^ pfu/mouse)	None	Tumor regression,prolonged survival, but not in T-cell-deficient mice	Effective retargeting elicits an effective tumor-directed T-cell response		[41]
Ad	ICOVIR-15 K	BiTA under major late promoter	ICOVIR-15 K-cBiTAto EGFR	8-week-old female SCID/beige mice	Lung cancer A549, Colon cancer HCT116	A549 tumors, IV 2 × 10^9^ VP; HCT116 tumors, IV 1 × 10^10^ VP			Enhances antitumor efficacy in vivo	OV-BiTA can overcome key limitations	Oncolytic properties reduced twofold	[43]
Ad	EnAd		BiTA to EpCAM	HEK293A, DLD, SKOV3, MCF7, A431, A549, NHDF and PC3, CHO	Multiple cancers	Cells incubated in 50% exudate in 500 ng/ml BiTA or 100 vp/cell EnAd			A marked cancer cell depletion	A new treatment of disseminated cancer		[101]
Ad	NSC.CRAd-S-pk7		NSC. Survivin promotor, a poly-L-lysine (pk7)		Ovarian cancer mice model	3 weeks of 1 × 10^6^ cells [5 × 10^8^ pfu]/day	Cisplatin	Not significantly worsen toxicity by daily score	More substantial decreases in omental tumor burden	Increased efficacy with no added toxicity	Its replication is conditional upon overexpression of survivin	[84]
Ad	DNX-2401 (Delta-24-RGD; tasadenoturev)	a 24 bp deletion in E1A	RGD-motif into the fiber H-loop	DIPG and pHGG cell lines	pHGG/DIPG mice model	Delta-24-RGD (10^8^ pfu/animal) intracranially 1 or 3 times in 3–4 μl 3 days later		No adverse effect	Increased survival by an average of 40 days (P = 0.024, Log-rank test)	Therapeutic option for pHGG and DIPG		[42]
Ad	oAd-MSCs			BALB/c mice	Renal adenocarcinoma, melanoma	2 × 10^6^ DiR-labeled oAd-MSCs per mouse, IP injected			Tumors decrease by 50% and inflammatory	TAMS and NK infiltrated, and TIL changed		[40]
VV	EphA2-TEA-VV		EphA2-TEA	SCID Beige mice; A549 cells	NSCLC	IP injection, 1 × 10^8^ pfu	PBMCs	None	Significant tumor growth decrease	The EphA2-TEA-VVs activated human PBMCs		[47]
VV	VVDD	hSNF5		CB17 SCID mice	AT/RT	50 μl VVDD-hSNF5 or VVDD GFP			Significant tumor regression	Cell cycle arrest and proliferation inhibit		[160]
MV	MV-H		DARPins	6- to 12-week-old female Hsd: Athymic Nude-Foxn1^nu^ mice	ovarian carcinoma	IP four times, 2 × 10^6^ TCID_50_/injection			The tumor burden reduced by 76% (MV-Ec4-Pro_9_-G3) to 95% (MV-Ec4)	harbor an intrinsic and robust specificity for heterogeneous tumor cells	DARPin/HER2 interaction inhibitsvirus spread	[48]
MV	MV-BiTA		MV-eGFP-mCD3xCEA	C57BL/6 J mice	Primary human colorectal cancer	intra-/peritumoral injection, 10^6^ pfu in 100 mL	BiTA to CEA		Increased T-cell infiltration and activation	Tumor-restricted continuous BiTA expression and in situ vaccination effects	OVs comparison lacking	[100]
Arenavirus	LCMV			MOPC-tumur-bearing C57BL/6 mice	Colon cancer, melanoma, hepatocellular carcinoma	2 × 10^4^ PFU peritumourally or 2 × 10^6^ PFU IV	CD8^+^ T cells; PD-1 blockade		Increased local and splenic virus propagation for more than 30 days	Effective tumor treatment	Not known in humans	[161]
Rhabdovirus	MG1		eGFP tagged Maraba	S180; 6 week old female Balb/C mice	Sarcoma	IT, 3 doses MG1 (1 × 10^8^ pfu/mouse) at days 8, 10 and 13			Eradication of 80% of tumors and protection from re-challenge	MG1 based oncolytic immunotherapy		[162]
NDV	NDV-ICOSL		NDV-ICOSL	Mice	B16-F10 melanoma	On days 7, 10, 13 and 16, IT, 100 μl of 2 × 10^7^ pfu	Anti-CTLA-4		Enhanced T cells infiltration and anti-tumor effect	A strong rationale for clinical evaluation	Mechanism not known. Subset patients	[108]
Canine virus serotype 2	ICOCAV17	E1ΔD21	human PH20 hyaluronidase *(PH20)*	Dogs	Spontaneous tumors	dCelyvir administered over 45 min through a peripheral or central venous line at 0.5 × 10^6^ cells/kg	i.v. with metilprednisone 1 mg/kg, metamizol 30, difenhidramine 0.5	27% (4) show clinical AE	74% response rate, 14.8% complete responses	OV-MSC represents an effective cancer therapy	Hyaluronidase for EMC	[44]
Coxsackievirus	CVA21			Peripheral blood mononuclear cells	AML MM	The PBMC exposed to CVA21 for 24 h			CVA21 stimulated potent anti-tumor immunity	AML cells resistant oncolysis, immune- killing of MM/AML observed		[163]
Myxoma virus	MYXV		IL-15 complex with a subunit of its receptor and tdTR	6–8-week-old C57BL/6 female mice	Melanoma	Injected (day 9) with a single dose of MSCs (5 × 10^5^/100 mL PBS)			Marked regression of lesions and could increase survival	MSCs ferrying MYXV to pulmonary melanoma foci triggering immune effects		[83]
Bovine pestivirus	BVDV			NOD-SCID mice	MM	IT twice a week for 2 weeks	bortezomib		significantly reduced tumor burden	BVDV has direct oncolytic effect in myeloma		[164]
Zika virus	ZIKV-Dakar	a 10-nt deletion in the 3’ UTR		C57BL6/J mice, 4 × 10^4^ GL261 or CT2A glioma cells	GBM	IT, mouse-adapted ZIKV (10^5^ FFU)	Anti-PD-1, IP on days 8, 10, 12, and 14, 10 mg/kg		Combination therapy improved long-term survival to 80%		Optimization of the timing of ZIKV administration	[27]

Recently, Lin et al. developed a novel immunotherapeutic HSV-1 (OVH-aMPD-1) expressing a scFv against PD-1, which releases damage-associated molecular patterns (DAMPs), promoting antigen cross-presentation by DCs, and enhancing the infiltration of activated T cells; these modifications resulted in activation of antitumor T-cell that led to reduced tumor burdens in a mouse model of liver cancer [29]. In addition to awaken T cell response, activating other types of immune cells is also a wise option. The combination of EGFR-CAR NK-92 cells with oHSV-1 resulted in more efficient killing of MDA-MB-231 breast tumor cells and significantly longer survival of tumor-bearing mice when compared to monotherapies [36]. A UV light-inactivated HSV-1 (UV-HSV-1) potently activates human peripheral blood mononuclear cells (PBMCs) to lyse leukemic cell lines and primary AML samples, but not healthy allogeneic lymphocytes. The data suggested that UV-HSV-1 synergizes with IL-15 and IL-2 in inducing activation and cytolytic activity of NK cells [37]. Moreover, to reduce toxicity and enhance oncolysis to destroy glioma, Delwar et al*.* replaced the HSV *ICP4* promoter with the *survivin* promoter and introduced the 5’UTR of rat FGF-2, and 5 copies of the miRNA 124 target sequence 3’UTR into the *ICP4* gene. The intratumorally injected oHSV-1 was demonstrated to be effective in mice bearing human glioma U87 tumors, whereas viral DNA was almost undetectable in normal organs [38]. To evade antiviral defense response, arming oHSV with antiangiogenic N-terminal cleavage fragment of brain angiogenesis inhibitor (Vstat120) shields oHSV-Vstat120 from inflammatory macrophage antiviral response, without reducing safety [39]. oHSV-Vstat120 treated mice harboring renal adenocarcinoma and melanoma tumors presented increased infiltration of tumor-associated macrophages (TAMs), NK cells, and tumor-infiltrating lymphocytes [40].

Activating the host immune system seems to be a popular route for potentiating anti-tumor effect of OVs. Polysialic acid (polySia) is expressed on several malignant tumors of neuroendocrine origin. PolySia-dependent systemic infection in vivo facilitated effective uptake of viruses in subcutaneous polySia-expressing human tumors, whereas hepatic viral load and hepatotoxicity were significantly reduced. Enhanced tumor regression and prolonged survival was only observed in immunocompetent mice, but not in T-cell-deficient mice, suggesting that a polySia-retargeted oAd elicits an effective tumor-directed T-cell response after systemic virus delivery and facilitates therapy of disseminated lung cancer [41]. DNX-2401 (Delta-24-RGD; tasadenoturev) is a tumor-selective, replication-competent oAds, which is proven to be safe in mice and results in a pronounced increase in survival in immunodeficient and immunocompetent models of high-grade pediatric glioma and diffuse intrinsic pontine gliomas [42]. The Ad was engineered to express an EGFR-targeting BiTA (cBiTA) antibody under the control of the major late promoter, leading to generation of ICOVIR-15 K-cBiTA, which bound specifically to both CD3 + and EGFR + cells. Intra-tumor (IT) injection of this cBiTA-expressing Ad increased the accumulation and persistence of tumor-infiltrating T cells and the antitumor efficacy in vivo [43]. Actually, as MSCs present tropism for tumors, the use of MSCs to transport OVs to tumor sites is a promising alternative to IT administration [40]. The data suggested that treatment with oAd-MSCs significantly reduced tumor volumes by 50% and induced a pro-inflammatory TME. In a veterinary dog trial with dCelyvir (canine MSCs infected with an oAd ICOCAV17) in 27 canine patients, Cejalvo et al*.* observed an excellent toxicity profile as well as a clinical benefit in 74% of patients, including 14.8% showing complete remissions [44]. Actually, it is a very promising attempt to arouse T cells by designing BiTAs OVs [45, 46]. Particularly, together with T cells a VV encoding a secretory BiTA consisting of two scFvs specific for CD3 and EphA2 (EphA2-TEA-VV) had potent antitumor activity in comparison with control VVs plus T cells in a lung cancer xenograft model [47]. In vivo, the therapeutic efficacy of MVs targeted to HER2/neu and EpCAM by designing ankyrin repeat proteins (DARPins), was confirmed in an orthotopic ovarian carcinoma model revealing an effective reduction of tumor mass [48]. Overall, these successful preclinical results have made a decisive contribution to further investigation in the clinics.

## Safety of oncolytic virotherapy

**Fig. 2:**
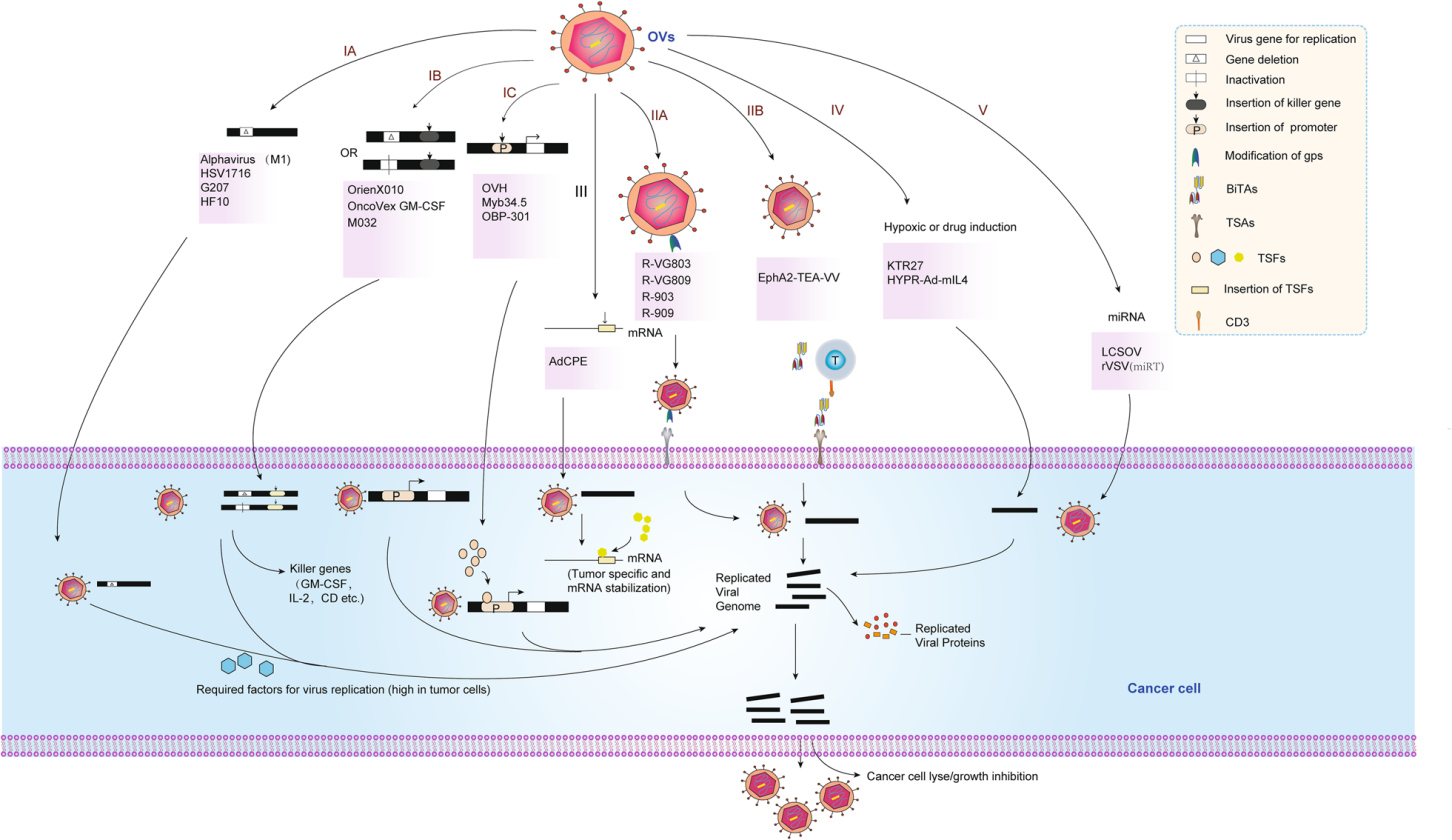
**The tumor specificity of oncolytic virotherapy.** IA: Deletion of the required genes for virus replication in normal cells. IB: Deletion or inactivation of the required genes for virus replication and insertion of killer genes. IC: Transcriptional targeting. IIA: Retargeting strategies based on genetic manipulations of glycoproteins. IIB: T-cell activator. III: Translational targeting. IV: Hypoxic or drug induction. V: Intracellular restrictions by miRNA targeting

Therapeutic safety remains a paramount concern during OVT while the tumor targeting/tropism is a highly desirable characteristic for OVs. Generally, tumor-specific and natural receptors were responsible for tumor selectivity and cell entry. To achieve cancer cell specificity in different OVs, a few viruses, e.g., parvovirus and NDVs, own a naturally tumor tendency. Many, if not most, such as MVs, Ads, VSVs, VVs and HSVs exhibit no preference for cancer cells. Thus, the viruses from these families need to be designed to preferentially target cancers rather than normal tissues.. Genetically engineered viruses can be exploited in several aspects, such as tumor cell receptor targeting, driving the expression of certain viral replication genes by promoters and enhancers, translational targeting, engineered microRNA target sequences, immunogenic tumor-associated antigen targeting, etc. (Table 2 and Fig. 2) [19, 49]. Taking HSV, one of the most widespread and widely used OVs, as an example, to improve its safety, various engineering and modifications have been carried out on its genome [24]. Mutants of HSV-1 with deletion of *ICP34.5* and *ICP47* genes (such as T-VEC) have been successfully harnessed as attenuated oncolytic vectors [50, 51]. For HSV-based OVT, the detargeting-retargeting strategies so far were based on genetic manipulations of glycoprotein (g) D, gB and/or gH [52]. In particular, to enhance the tumor tropism and safety of HSV, a novel ligand in gH was designed to confer tumor cells entry [14]. To re-target the virus tropism to the HER2- and GCN4R-positive cells, the HER2 binding peptide was inserted in gB and GCN4 peptide in gD or gB [53, 54]. A safe and effective therapeutic oncolytic HSV-2 (deletion of *ICP47* and *ICP35.4*) was also be used in combination with doxorubicin for breast cancer treatment [55]. Similarly, arming the miR-122a complimentary sequences to HSVs have shown high specificity to target hepatocellular carcinoma cells [12]. Engineering miRNA target sequences into viruses’ genomes was thereby inhibiting spread in tissues expressing cognate miRNAs. Tumor-specific translational regulation presents an attractive possibility for generating oncoselective therapies. Villanueva et al*.* reported the insertion of CPE regulatory sequences in the 3’-UTR of the *E1A* gene that confers translational E1A expression regulation, resulted in tumor-specific AdCPE viruses [56]. It is demonstrated that neurotoxicity was most profoundly reduced in a virus carrying four tandem copies of a neuronal miR-125 target sequence inserted in the 3′-UTR of the VSV polymerase gene [57]. Alexander Muik et al*.* have engineered a chimeric VSV, an oncolytic virus called rVSV (GP) devoid of natural neurotoxicity with undetectable immunogenicity and enhanced oncolytic potency [58].

**Table 2:** Tumor specificity mechanisms of OVs

Mechanism type	Virus	Viral gene and its modification	Viral gene function	Mechanism or target protein	Comments and advantages	Unresolved issues, problems or disadvantages	Refs
Transcriptional targeting	HSV-1	oHSV1-SS1, Signal-Smart 1 (SS1). ICP4 expression under ELK	ICP4, a viral protein necessary for replication	oHSV1-SS1 infects only host cells with overactivation of the Ras/ERK/ELK pathway	SS1 virus preferentially infects prostate cancer cells and induces changes in viability, invasiveness and necrosis	ELK signaling may not reflect the situation in tumor tissues	[165]
Transcriptional targeting	HSV-1	HCC-specific gene promoters	Specific promoters drive selective viral gene expression	Transfer therapeutic genes; target, multiply in, and eradicate hepatoma cells via their lytic cycle	Some HCC-specific gene promoters were identified and can be used for virotherapy	The viral replication relies on the overexpression of B-myb in tumor cells	[11]
Transcriptional targeting	HSV-1	KTR27. The *tetR* gene controlled by the ICP0 promoter at the ICP0 locus and the essential ICP27 gene under the control of the *tetO*-bearing ICP27 promoter	ICP0 is required for viral gene expression, replication at low MOI and reactivation; ICP27 is an essential IE protein that modifies and transports viral transcripts to the cytoplasm	Repression of the *tetO*-bearing ICP27 promoter by *tetR* would greatly impair the ability of the virus to initiate productive infection in the absence of tetracycline	KTR27 can limit its replication to the targeted TME with localized tetracycline delivery, thus minimizing unwanted viral replication in distant tissues following local virotherapy	Whether KTR27 would be equally effective against small-cell lung cancer or NSCLC xenografts remains to be determined	[166]
Transcriptional targeting	Ad	HYPR-Ad-mIL4, The Ad E1A viral replication and IL-4 genes under the hypoxia/HIF-responsive promoter	Ad *E1A* makes cells more susceptible to virus replication	Bidirectional tumor-restrictive hypoxia/HIF promoter to drive viral *E1A* gene expression	Hypoxia-dependent IL-4 expression, viral replication, and conditional cytolysis of hypoxic cells	Limited to tumors that develop hypoxia/HIF activation	[167]
Transcriptional targeting	Ad	Telomelysin (OBP-301); hTERT promoter; combined with chemotherapy drugs: cisplatin and paclitaxel	hTERT promoter to express the viral gene; chemotherapy drugs	Drive the expression of *E1A* and *E1B* genes linked with an *IRES*, induces selective E1 expression, and selectively kills human cancer cells	Most cancer cells express Telomerase transcription factor	These findings need further research in vivo and in different tumor type to determine its validity	[122]
Transcriptional targeting	VV	rVACV is based on the tet operon of transposon Tn10	Tet operon can be activated tetracycline derivatives such as doxycycline	Exogenous control of gene expression levels by administration of a nontoxic inducer	The control of viral gene expression can benefit the safety of virotherapy	Induction rates need increase and the background expression need decrease	[123]
Transductional targeting	VSV	Replication-defective VSV, deleted its glycoprotein gene, VSVΔG, pseudotyped with MV-F and MV-H displaying scFv specific for EGFR, FR or PSMA	VSV G gene encoding VSV-G protein, for cell entry	The retargeted VSV (VSVΔG pseudotypes) infected only cells that expressed the targeted receptors (EGFR, FR, or PSMA)	Pseudotyped VSV infects only cells expressing the corresponding receptor both in vitro and in vivo	The prevalence of preexisting anti-measles antibodies in the patient population could neutralize the systemically administered virus	[168]
Transductional targeting	HSV	scFv-HER2-gH	gH/gL and gB constitute the conserved fusion apparatus	Engineering in gH of scFv directed to the cancer-specific HER2 receptor	Entry of viruses in the absence of gD or upon deletion of key residues in gD for the nectin1/HVEM binding	It can only be used for the tumor cells with HER2 receptor	[14]
Transductional targeting	HSV	gB-scFv-HER2	gB contributes to determine the virus tropism	Engineering in gB of scFv directed to the cancer-specific HER2	Activation of the chimeric gB-HER2 did not require the activation of the gD and gH/gL	Re-targeted to the HER2-positive cancer cells	[169]
Transductional targeting	HSV	gD-GCN4R and gD-HER2	Determine the virus tropism	Simultaneous insertion of both the GCN4 peptide and the Her2 scFv in gD	Re-targeted to the HER2 and GCN4R positive cells	Restricted to HER2 and GCN4R positive cells	[54]
Transductional targeting	HSV	gB-GCN4R and gD-HER2	Determine the virus tropism	Insertion of the GCN4 peptide in gB and detargeting plus HER2-retargeting via gD	Optimize the retargeted oncolytic HSVs to the translational phase	Restricted to the HER2 and GCN4R positive cells	[53]
Transductional targeting	SVV	Wild type virus	Anthrax toxin receptor 1 (ANTXR1)	SVV interacts directly and specifically with ANTXR1	ANTXR1 as the high-affinity cellular receptor for SVV	Non-modified virus	[170, 171]
Immune evasion	HSV-2	Δ *ICP47* and Δ*ICP34.5*	*ICP34.5*, a neurovirulence gene; ICP47 blocks MHC I function in infected cells	Δ ICP34.5 restricts oHSV replication to tumor cells and Δ ICP47 to promote virus oncolytic activity by up-regulating US11 and TAA presentation	Treatment with DOX followed by the oHSV2 was significantly more beneficial than treatment with either agent alone	The extracellular matrix restricts the initial distribution and subsequent spread of viruses in the tumor mass	[55]
Immune evasion	ZIKV	ZIKV-E218A, NS5 (E218A)	NS5 (E218A) has 2'-O methyltransferase activity	ZIKV-E218A sensitizes the virus to translational inhibition by type I IFN and IFIT1	Lysis of glioblastoma stem cells (GSCs) with less toxicity to normal neural cells	The anti-tumor effect remains to be determined n patient-derived GSCs in vivo	[172]
Immune stimulation	NDV	NDV-expressing ICOS ligand (NDV-ICOSL)	Enhance systemic immune checkpoint blockade	NDV-ICOSL enhances tumor control, TIL infiltration, the efficacy of CTLA-4 blockades	Potentially avoiding additional systemic toxicity	ICOSL could have additional interaction partners	[108]
Immune stimulation	Ad	a 24-base-pair deletion in the *E1A* gene (Ad5D24)	*E1A* makes cells more susceptible to virus replication	Ad coated with MHC-I tumor epitopes (the modified poly-K-SIINFEKL, PeptiCRAd)	significantly improve the response rate to checkpoint blocking antibodies		[78]
Post-transcriptional targeting	Ad	Insertion of CPE regulatory sequences in the 3’-UTR of the *E1A* gene (AdCPE)	*E1A* makes cells more susceptible to virus replication	CPEB4 bind to CPEs in the 3’-UTR of *E1A* confers E1A expression post-transcriptionally, resulted in tumour-specific oHSV	CPEB-dependent regulation can be exploited to attenuate viral toxicity, by preventing the spread of the virus in normal tissues	Rely on the cellular transcription machinery, but not for viruses that use virally encoded polymerases in the cytoplasm, such as the MV and VV	[56]
miRNA targeting	VSV	4 tandem copies of a neuronal miRNA125 target sequence inserted in the 3’-untranslated region of the viral *polymerase (L)* gene	*Polymerase L* gene coding for RNA-dependent RNA polymerase	miRNA125 targets engineered into VSV to ameliorate its neuropathogenicity by restricting viral replication in specific tissues	Compared to picornaviruses and adenoviruses, the VSVs were relatively resistant to miRNA-mediated inhibition, but neurotoxicity was ameliorated significantly	Mutation and selection of viruses containing altered miRNA target sequences could be a potential pitfall, with mutations in the miRT sequence reducing the efficiency	[57]
miRNA targeting	HSV	apoE-AAT promoter linking with gH and miR-122a complimentary sequence at 3’UTR of gH (LCSOV)	gH is needed for virus assembly and cell entry	Viral gene are replicatible in HCC owning to absent of miR-122a	LCSOV is a safe oHSV that can precisely target HCC both in vivo and in vitro	The strategy depends heavily on promoter activity in the targeted tumor cells	[12]
Translational targeting	HSV-1	ICP6 expression is defective, and expression of the HSV-1 γ1 34.5 gene is regulated by the cellular B-myb promoter (Myb34.5)	The *UL39* gene encodes ICP6, an ICP6 mutant HSV that can only replicate in dividing cells	oHSV γ1 34.5 kills tumor cells by PKR-induced inhibition of cell proliferation and tumor growth; ICP6 defective oHSV efficiently replicates and kills dividing cells	HSV-1-based selective Myb.34.5 virus effectively replicates and kills PDAC-derived cells both in vitro and in vivo	The viral replication relies on the overexpression of B-myb in tumor	[13]

Here we summarized the virulence and tumor specificity mechanisms of different virus families in recent years (Table 2). Among them, the selection of tumor-specific antigens is a leader in increasing the safety of OVs. The detargeting-retargeting strategies were based on genetic manipulations of glycoprotein of different types of viruses, such as antigens of HER2, EGFR, GCN4, EpCAM have been sucessfully applied in HSV, VSV and MV etc. To date, OVT have been evaluated for safety by both localized and systemic administration. The most common adverse effects are fever and general flu-like symptoms. Moreover, no transmission of OVs from treated patients to others has been reported [19]. However, therapeutic safety concerns must be scrupulously addressed to ensure the safety of patients and other people who may have contact with the patients. The development of OVT were greatly benefited from the studies on structures and characteristics of virus particles [59–62]. More engineered OVs for particular tumor treatment will be safely applied in clinical trials and approved protocols.

## Efficacy of oncolytic virotherapy

**Fig. 3:**
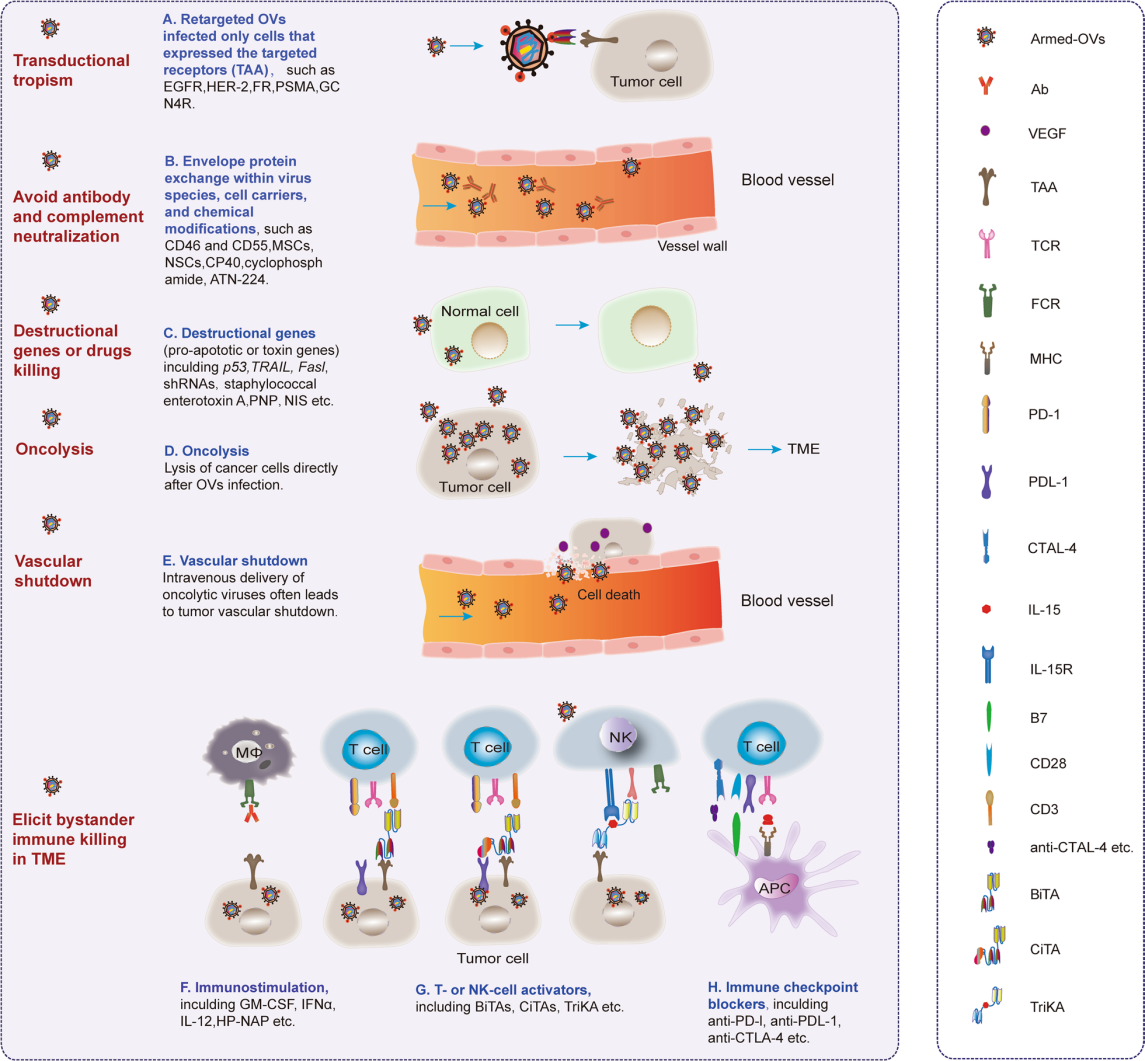
The therapeutic efficacy of oncolytic virotherapy

Although safety concern is a paramount priority, high efficacy to eliminate tumors is the goal of OVT. OVs can destruct cancer cells in many ways, including direct oncolysis, antitumor immunity, vascular-disrupting effect, bystander killing effect [17]. Therefore, to pursue the ideal therapeutic effect, we may start from following aspects. First, the importance of tumor targeting in improving therapeutic effect is out of question. Due to the rapid replication and cell lysis properties of some virus families, with a wide range of tissue tendencies, it is necessary to continue rational optimization of these viruses to efficient kill specific types of cancer. For example, the natural neurotropism of HSVs has made it attractive as vectors for the development of OVs for application in the nervous system [63, 64]. Moreover, retargeted OVs infected only cells that expressed the targeted TAAs, such as EGFR, HER-2, PSMA, GCN4R (Fig. 3A and Table 3). Second, suitable doses and delivery system of OVs in administration, such as intratumor (I.T.), intra-vein (I.V.) and intra-muscle (I.M.) injection, are required [65–67]. Third, to elicit the bystander immune response is a preeminent blueprint [68, 69]. Fourth, arming the viruses with destruction/immunostimulatory genes, innovative combination with other therapies are promising strategies gaining momentum [70]. Herein, the arming mechanisms of OVs were summarized (Table 3 and Fig. 3) and discussed below.

**Table 3:** The therapeutic efficacy mechanisms of OVs

Efficacy mechanism	Virus	Gene	Gene function	Mechanism or target protein	Comment, advantage	Unresolved issue, problem or disadvantage	Refs
NAb evasion	Ad	Ad5; Ad5-RGD; Ad5/3	Avoid NAbs	Fiber knob modification	Avoid the NAb response in human cancer patients	NAb is not the only anti-viral defense system	[88]
NAb evasion	MV	TRMV ectodomain	Avoid NAbs	The MV F cytoplasmic tail and a TPMV H protein with a truncated cytoplasmic tail	Avoid the MV-neutralization	Lost some fusion function	[76]
NAb evasion	VSV	LCMV-GP	To abrogate neurotoxicity, circumvent humoral immunity	rVSV (GP) escapes humoral immunity	The neurovirulence of VSV is mitigated Avoid the inactivation by complement and NAbs	Not occur naturally, preclinical safety assessments must be extensive and thorough	[58]
Complement evasion	NDV	CD46, CD55 in the viral envelope	To enhance complement evasion	Regulators of complement activity (RCA)	To enable the NDV to resist the complement	Homologous restriction	[89]
Complement evasion	VV	Pexa-Vec; complement inhibitor, CP40	CP40 inhibits the function of complement	The complement dependence of anti-vaccinia antibody	CP40 enhance the delivery efficacy of virus	No AE was not observed	[90]
Cancer cell and CAF interaction	VV, VSV∆51, Maraba MG1 virus	FGF2	To prevent the ability of malignant cells to detect and respond to virus	TGF-β produced by tumor cells reprogrammed CAFs. CAFs produced FGF2 to reduced retinoic acid-inducible gene I (RIG-I) in cancer cells	OV encoded to produce FGF2 is safe in tumor-bearing mice and show improved therapeutic efficacy	The specific molecular mechanism remains to be elucidated	[121]
Cell carriers	MV	MSC	MSC transferred MV infection to target cells	The protection from anti-measles antibodies, preferentially accumulate at tumor sites	Cell carriages protect MV from the effect of neutralizing antibody	MV infected MSC did not produce a significant amount of progeny virus	[79]
Cell carriers	HSV	MSC	MSC in sECM, then used for the tumor lesions	Killing of GBMs in vitro and in vivo by oHSV infection and tumor destruction	sECM-encapsulated MSC-oHSVs result in statistically significantly increased anti-GBM efficacy	The conventional GBM cell lines used here	[115]
Cell carrier	HSV	MSC	Intra-arterial delivery of MSC-oHSV can effectively tracks and kill metastatic tumors	Effectively metastatic melanoma cells in the brain, and that combination therapy with an immune checkpoint blocker boosts the efficacy	Overcomes the hurdles of systemic delivery	Need MSCs	[72]
Cell carrier	Ad	BM-hMSCs	Intraarterial delivery effectively eradicated human gliomas	Delta-24-RGD infects and replicates in PD-BM-hMSCs, that PD-BM-hMSCs effectively deliver Delta-24-RGD to the tumors	Overcomes the hurdles of systemic delivery	Need BM-hMSCs	[81]
BiTA	VV	EphA2-TEA-VV	Redirecting T cells to tumors	Killing of viral infected and noninfected tumor cells, “bystander killing”	Improved antitumor T-cell responses	The complete clinical responses rarely observed	[47]
BiTA	Ad	EnAdenotucirev (EnAd) EpCAM-CD3	BiTA to EpCAM	BiTA leads to clustering and activation of both CD4 and CD8 T cells; BiTA under the virus major late promoter	Activation of endogenous T cells to kill endogenous tumor cells despite the immunosuppressive environment	Limited to EpCAM-positive tumors	[101]
BiTA	Ad	ICO15K-cBiTA. E2F binding sites and an RGDK motif	cBiTAs to EGFR + cells	Increased the persistence and accumulation of tumor-infiltrating T cells in vivo	Robust T-cell activation, proliferation, and bystander cell-mediated cytotoxicity. Shown favorable toxicity profiles	The oncolytic properties reduced twofold compared with the nonmodified virus; Limited to EGFR-positive tumors	[43]
Immune stimulation	HSV	GM-CSF	Stimulates the production and maturity of immunocytes		HSV can inhabit the growth of pancreatic carcinoma	The agent was highly attenuated	[98]
Immune stimulation	HSV-1	GM-CSF	Local and systemic anti-tumor response	A rapid eradication of malignant cells and Enrichment in cytotoxic T cells and a decrease of regulatory T cells in injected and noninjected lesions	Interferon pathway activation and early influx of natural killer cells, monocytes, and dendritic cells	T-VEC HSV proteins in FNA and immunohistochemistry needed. Functional viral replication in nonmalignant cells needed	[112]
Immune stimulation	HSV-2	Deletion of ICP34.5 and ICP47	ICP34.5 is a neurovirulence gene; ICP47 blocks antigen presentation		The oncolytic activity of HSV-2 is like HSV-1 and can be improved by the sequential use of doxorubicin	Physical barriers restrict the initial distribution and subsequent spread of viruses	[55]
Immune stimulation	HSV	G47Δ-mIL12	IFNγ and T cell killing inducers	Induces M1-like polarization (iNOS + and pSTAT1 +) in TAMs	The synergistic interaction between G47Δ-mIL12 and two checkpoint inhibitors (anti-CTLA-4 and anti-PD-1) in curing glioblastoma and inducing immune memory	Multiple distinct immunotherapeutic strategies will likely be required	[32]
Immune stimulation	HSV	Ruxolitinib (RUX). Δγ34.5	Constitutively activate STAT signaling	Ruxolitinib improved viral replication and immune response	Increased CD8 + T-cell activation in the tumor microenvironment		[31]
Immune stimulation	VSV	lipopolysaccharide (LPS)	LPS, a TLR-4 agonist, activating innate immune response		LPS can enhance the local therapy effects induced by IT treatment of VSV		[87]
Immune stimulation	Ad5	Helicobacter pylori neutrophil-activating protein (HP-NAP)	HP-NAP can recruit neutrophils and induce Th-1 type differentiation		HP-NAP improves the anti-tumor effect through the activation of innate immune system	The systemic level of HP-NAP cannot be measured	[120]
Immune stimulation	VV	HPGD	HPGD is a prostaglandin 2 (PGE2) inactivating enzyme	Reduce MDSC, re-sensitize resistant tumors, enhancing systemic attraction of T cells	HPGD targets PGE2 and depletes G-MDSC; Alters chemokine profiles and immune cell infiltrate	Inducing inflammation, unable to prime adaptive immunity	[173]
Immune stimulation	NDV	NDV-ICOSL	ICOS ligand targets ICOS-positive tumor	Enhanced infiltration with activated T cells, and effiency together with systemic CTLA-4 blockade	Combination therapy leads to the expansion of activated TILs	The optimal pathways not known; Limited to a subset of patients	[108]
Immune stimulation	poliovirus/rhinovirus chimera	PV receptor CD155	CD155 is a ligand for CD226, TIGIT, and CD96 with roles in immune response modulation	Stimulates canonical innate anti-pathogen inflammatory responses within the TME that culminate in dendritic cell and T cell infiltration	In addition to lytic damage to malignant cells, noncytotoxic infection of APCs/DCs involved	The use of murine models and in vitro systems, not in patients	[111]
Immune stimulation		CD28	CD28 provide co-stimulatory signals, which are required for T cell activation	Highlight intratumoral CD28 co-stimulation by myeloid-antigen-presenting cells for activation of PD-1 + tumor-infiltrating T lymphocytes during PD-1 blockade in HGSOC	Optimal tumor-specific T cells required for immunotherapy	Not address the immunologically ‘‘cold’’HGSOCs. Some of these tumors completely lack recognition of TAAs by T cells, whereas others simply exclude the tumor-specific T cells from TME	[113]
Apoptosis	HSV-2	Her2-COL-sFasL	sFasL-containing molecules induce cell apoptosis	Secretable and self-multimerizing sFasL improved the potency	The bystander effect through the tumor cell apoptosis	Cause the death of normal cells	[17]
Apoptosis	HSV	oHSV-TRAIL	Alters cell proliferation, death and DDR pathways	Inactivate MEK/ERK and Chk1 signaling pathways, which underlies the anti-GSC activity of oHSV-TRAIL	Potent therapeutic efficacy of an apoptotic variant in glioblastoma models that recapitulate chemo-resistance and recurrence		[158]
Transductional targeting	Ad	Ad-hTERT, CARsc-pSia	Highly polySia-selective retargeting	A bispecific adapter comprising the coxsackievirus/adenovirus receptor ectodomain and a polySia-recognizing scab	Elicits an effective tumor-directed T-cell response after systemic virus delivery and facilitates therapy of disseminated lung cancer	Limited to CAR-deficient, polySia-positive cancer	[41]
Transductional targeting	HSV	oHSV-scFv-HER2 (R-LM113) or HSV-scFv-oHER2-mIL-12 (R-115)	IL-12 to elicit a local immune response scFv to HER2	R-115 unleashed the immunosuppressive tumor microenvironment	A reduction in the growth of the primary and distant tumor	Limited to HER2-positive cancer	[174]
Transcriptional targeting	HSV-1	ICP6 defective. γ_1_34.5 under B-myb promoter	γ_1_34.5 protein can circumvent the consequences of PKR activation	Myb34.5 replicates to high level in human PDAC cell lines and is associated with cell death by apoptosis	Virus replicate to high level selectively in PDAC cells	Limited to B-myb present	[13]
DARPins	MV	DARPins	Targeted both to HER2*/neu* and EpCAM	Simultaneously targeted to tumor marker HER2/neu and CSC marker EpCAM	High in vivo efficacy with the potential to handle IT variation of antigen expression	The CSC targeting remains to be elucidated	[48]
PARP*i*	HSV	PARP*i*	Targeting DDR in cancer with HR repair deficiencies	Increased sensitivity to PARP*i* due to oHSV-induced Rad51 loss	Overcomes the clinical barriers of PARP*i* resistance and DNA repair proficiency	The large diversity between different patient GSCs genomically	[33]
NIS	MV	Thyroidal sodium-iodide symporter (NIS)	Monitoring by noninvasive imaging of radioiodine	CD46, which is the cellular receptor for MV-NIS, mediating both virus entry and subsequent cell killing through cell–cell fusion	MV-NIS can replicate before being cleared by the immune system. Monitored non-invasively	The small sample size of patients treated in phase II trial	[150]
Prodrug activation	Reovirus-3	RT3D. Drug: cyclophosphamide	Improve viral delivery by immune suppression	Cyclophosphamide may improve tumor delivery	Administration with the association of PBMCs may enhance effiency	Cyclophosphamide is ineffective in this clinical trial	[85]
TGF-βR inhibitor	HSV	TGF-βR inhibitor	TGF-β drives, invasion/migration, angiogenesis, immune-suppression	Synergistic in killing recurrent GSCs through, JNK-MAPK blockade and increase in oHSV replication	A novel synergistic interaction of oHSV therapy and TGF-β signaling blockade	1) treatment at an early time-point, 2) the use of a nodular GBM model	[156]
Immune checkpoint inhibitor	VV	PD-1/PD-L1 blockade	Enhances virus-specific CD8^+^ T-cell responses and reduced viral load	Dual therapy elicited systemic and potent anti-tumor immunity。	Eliminated immunosuppressive cells (including MDSC, TAM, T*reg* and exhausted CD8 + T cells), and elicit more anti-tumor immunity	The toxicity; VV elicited a host antiviral immune response, and immune suppressor cells recruitment	[175]
Virus stability	HSV	ATN-224	ATN-224 can form chelate with copper ion	ATN-224 increased serum stability of oHSV and enhanced the efficacy of systemic delivery	Greatly enhanced its replication and antitumor efficacy	The specific mechanism needs further study	[86]
Chemokine	HSV-2	FusOn-H2. Deletion of ICP10 protein kinase domain	Viruses attract T cells to the infected tumor cells	Improve the therapeutic effect through the high level of chemokines in the tumor lesion	Combined with adoptive T-cell therapy	The specific mechanism has not been clarified	[176]
Immune evasion	HSV	BAI1, and its N-terminal cleavage fragment (Vstat120)	Vstat120 inhibits TNFα production by blocking BAI1-mediated macrophage response	Reduced macrophage/microglial infiltration, activation and TNFα production	Shields from inflammatory macrophage antiviral response without reducing safety	How Vstat120 might block the function of BAI1 is currently unclear	[39]
CDH1	HSV	CDH1	E-cadherin, a ligand for KLRG1, an inhibitory receptor on NK cells	E-cadherin enhanced the spread of oHSV-CDH1 by facilitating cell-to-cell infection and viral entry and reduced viral clearance from NK cells	Simultaneously blocks cytolytic NK cell activity and promotes viral infectivity	Just blocks NK cells	[177]
RNA interference	HSV-1	Bcl-2 and Survivin RNAi sequences	The knockdown of Bcl-2 and Survivin genes	Improves the antitumor effect of OVs in high PKR phosphorylation tumor cells	Dual silencing of Bcl-2 and Survivin improved the antitumor effect of oncolytic HSV-1 in vitro and in vivo	In the low PKR phosphorylation tumor cells, the antitumor effect is restricted	[118]

The optimization on virus spread and delivery of OVs play a crucial role directing therapeutic efficacy. There are several host barriers hampering the potency of OVT in patients. If the OVs is not administrated I.T., I.V. and I.M. injection of OVs was usually hindered by antibodies and complements in the blood stream. Thus, it is essential to develop strategies to escape antibody and complement neutralization in the blood stream. To limit the neutralization of OVs, there are several classical oncolytic vector shielding strategies, including envelope protein exchange within a virus species or families, multiple epitope replacements, devising cell carriers, and chemical modifications [19, 49, 71, 72].

To restrict antibody-mediated HSV neutralization, the antibodies targeting functional epitopes on HSV glycoproteins can mediate neutralization directly. For example, epitopes modification on HSV have been well-defined and characterized in humans [73–75]. MVs-based shielded oncolytic vectors to circumvent antibody neutralization have been developed by exchanging the envelope glycoproteins, hemagglutinin (H) and fusion (F) protein, with those from the non-cross-reactive Tupaia paramyxovirus [76]. In genital disease, HSV-2 vaccination with human papillomavirus vectors expressing HSV glycoprotein antigens was developed successfully for eliciting anti-viral response [77]. Cristian et al. demonstrated that Ads coated with MHC-I tumor epitopes increase the antitumor immunity and efficacy against melanoma [78]. Cell carriers, such as cytokine-induced killer cells, mesenchymal stem cells (MSCs), neural stem cells (NSCs), and stromal vascular fraction cells (SVFs), are capable of accelerating the OVs delivery to tumors and in the same time protecting OVs from antibody neutralization [7, 79–81]. Multiple studies have demonstrated that MSCs or NSCs allow for safe and efficient ferrying of OVs to tumor foci to trigger immune response [65, 71, 79, 82–84]. Specifically, a TK-positive oVV ACAM2000, delivered by autologous adipose SVF cells, fostered such treatment in the patients with advanced solid tumors or acute myelocytic leukemia (AML) in a great safety and accessibility. The clinical data revealed that the viral DNA could be readily detected in all patients’ blood samples immediately after treatment [80]. Certainly, chemical or other modifications are also good OVs shielding option. Reoviruses and HSVs have been armed with cyclophosphamide, an immune modulator, to combat the antibody neutralization, thereby enhancing the virus infection [85]. Since copper in serum prevents replication of HSV-1, when armed the oHSV with a copper chelator ATN-224, significantly enhanced its therapeutic efficacy by increasing serum stability and systemic delivery of oHSV [86]. Rommelfanger et al. have demonstrated that the combination of VSVs and LPS generated significantly enhanced therapy of melanoma B16ova tumors upon direct I.T. administration [87]. Besides, the modification of the fiber knob and an arginine-grafted biodecomposible polymer arming were proved to be a feasible strategy to dodge antibody neutralization during systemic administration [88]. When measured just before the second treatment cycle, serum neutralizing antibodies titers differed in 83% of patients, suggesting that even minor changes in the fiber knob would able to circumvent host antibody neutralization [88]. Another example of modification is that the NDVs armed with regulators of complement activity CD46 and CD55 could enhance the efficient complement evasion [89]. Some complement inhibitors, such as CP40, have been shown to abolish host antibody neutralization and augment the dose of infectious oVVs ferried to tumor sites [90].

**Fig. 4:**
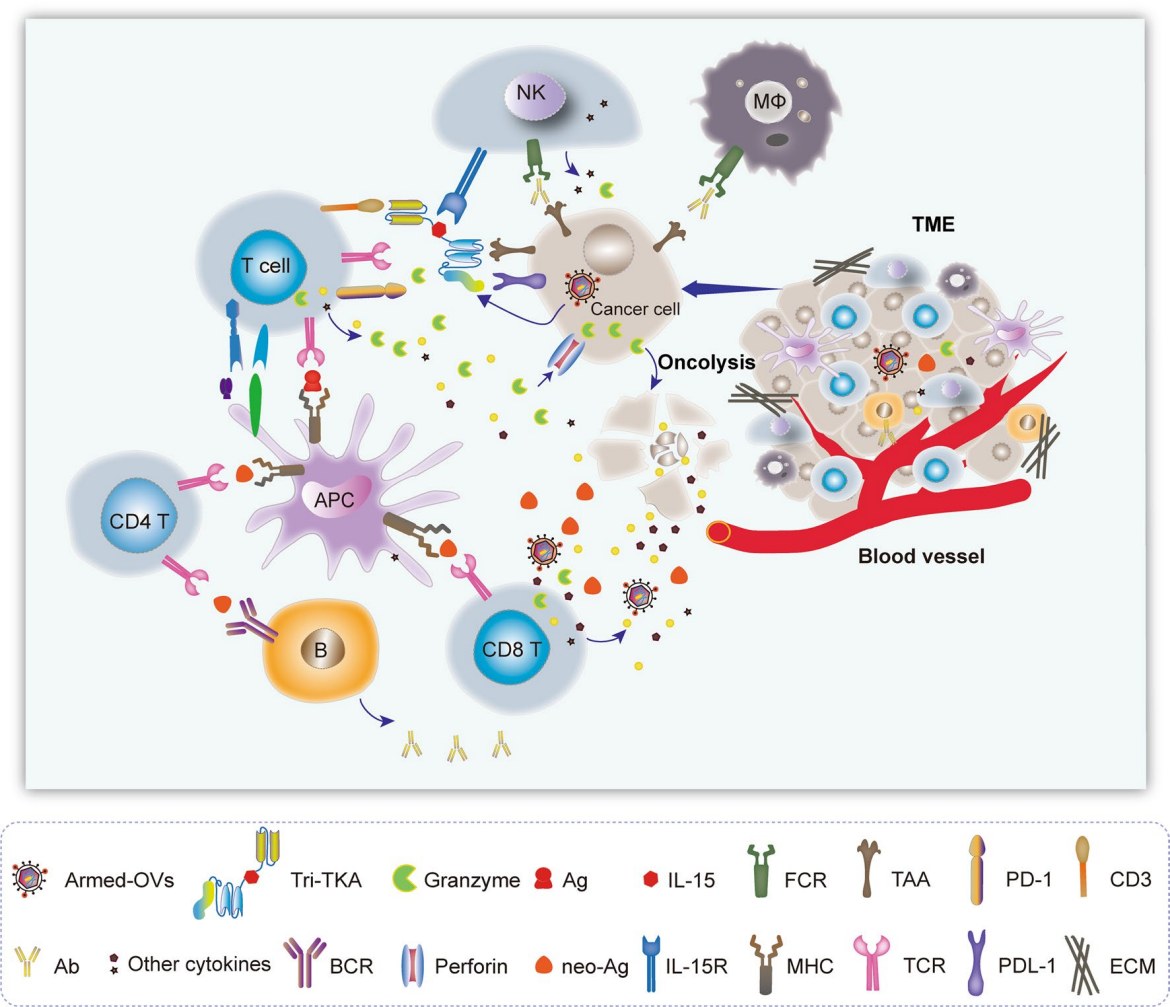
OVT in the tumor microenvironment (TME)

Once high doses of the viruses were maintained in the tumor microenvironment (TME), the therapeutic efficacy will be ultimately determined by the potency of OVs. As shown in Figs. 3 and 4, to reinforce the antitumor activity of OVs, eliciting bystander cell killing, introduction of pro-apoptotic or toxin genes and innovative combination therapy strategies were developed. OVs could use oncolysis to kill the infected tumor cells directly in TME. Except tumor cells, OVs can target several other components including cancer-associated fibroblasts (CAFs) and vascular endothelial cells (ECs). Then OV infection and the lysed cells causes the release of cytokines or neo-antigens, as well as the OV-armed immuno-stimulation genes, including GM-CSF, INF-γ, to initiate anti-viral immune priming by stimulating immune cells, including T cells, NK cells. The recruitment and maturation of innate immune cells which can cross-present TAAs to CD8 T cells, thus generating populations of TAA-specific CTLs. The generation of an OV infection-mediated anti-tumor immune response also counteracts the immunosuppression associated with myeloid derived suppressor cells and Tregs. In addition, the various destructive genes (such as pro-apoptotic genes, toxin genes) that are engineered within the OVs will take effects in TME. It is effective to mediate T and/or NK cell bystander killing of uninfected tumor cells in TME by engineering BiTA, CiTA, TriKA etc. (Fig. 4). Thus, OV infection acts on both the innate and adaptive immune system, which work together to kill cancers. The promising methods to create the bystander killing were prodrug activations, radiosensitization and immunostimulation [19]. For example, the purine nucleoside phosphorylase (PNP), one of convertase enzymes expressed in infected cells could convert prodrugs within the TME into toxic metabolites which eventually diffuse into and destruct adjacent uninfected tumor cells [19]. The sodium-iodide symporter (NIS) concentrates radioactive ions in infected cells, which triggers radiation poisoning of uninfected bystander tumor cells [19, 91, 92]. The clinical study demonstrated that oMV therapy can function as an antigen agnostic vaccine, increasing cytotoxic T-lymphocyte responses against TAAs in patients with multiple myelomas [92]. Of course, the most exciting strategy is the clinical application of OVs immunotherapy. The successful introduction of the granulocyte macrophage colony-stimulating factor (*GM-CSF*) gene into oHSVs represents a great breakthrough of immunostimulation. Such oHSVs, including T-VEC, CG0070, JX594, JX963, etc., have been shown in clinical trials to stimulate granulocytes and monocytes to elicit impressive anti-tumor immunity [21, 30, 93–95]. T-VEC, which produce GM-CSF, can efficiently treat the patients with metastatic melanoma, pancreatic carcinoma etc. [18, 21, 30, 96–98]. The phase III trial proved that local intralesional injections with T-VEC in advanced malignant melanoma patients can not only suppress the growth of injected tumors but also act systemically and prolong overall survival (OS) [30, 99]. Besides of immune stimulatory cytokines GM-CSF, IFNα, IL-12, IL-15 etc., immune checkpoint inhibitors (ICIs), bispecific T-cell activators (BiTA), some pro-apoptotic or toxin genes and shRNAs (targeting *Bcl-2, Survivin, COX-2* or *STAT3*) were also engaged in OVT [22, 29, 100–104]. The redirecting of T cells to the tumor by arming oVVs with BiTA (EphA2-TEA-VV) has the potential to boost the antitumor activity of oncolytic VVs [47]. An HSV-2 based OV can actively recruit T effector cells to the site of infection, suggesting that oHSV-2-based virotherapy can be armed with adoptive T-cell therapy to advance its therapeutic effect against solid tumors [105]. Expression of cytokines together with BiTAs has shown to mediate T cell bystander killing of uninfected tumor cells not only in vitro, but also in vivo [47, 100, 101, 106]. A combination of trans-genes encoding BiTAs, ICIs and APC enhancers will remove suppressive hurdles in the TME and allow for optimal antitumor efficacy of armed OVs [22]. The antibodies against immune checkpoint receptors have been exploited to conquer cancer by inducing T cell response, such as the antibodies against CTLA4, PD-1, PDL-1 and some alternative antibody formats (scFvs, Fabs, scAbs and VHHs) [22, 29, 107]. Zamarin et al. boosted the efficacy of systemic immune checkpoint blockade and avoided additional systemic toxicity by engineering a recombinant ICOS ligand-expressing NDV (NDV-ICOSL) [108]. Antibodies against immune checkpoint receptors, such as anti-CTLA4 and anti-PD-1, has clearly proven the therapeutic potential of antigen presentation and T-cell response against cancer [22, 29]. Moreover, the larger natural antibodies are not easy to eliminate and penetrate into solid tumors, the alternate antibody forms such as scFvs, Fabs, scAbs and VHHs have been increasingly exploited and applied [22, 29].

Maria et al. engineered a specific oncolytic Ads expressing a scFv of an antibody against PD-L1 to combine blockage of PD-1/PD-L1interaction with the antitumoral activity of Ad5 [109, 110]. They also armed Ads expression of an Anti-PD-L1-scFv improves anti-tumoral efficacy in a melanoma mouse model [109] Anthony et al*.* engineered the OVs to express a nonsignaling truncated CD19 (CD19t) protein tumor-selectively, enabling CD19-CAR T cells to target, and showing effective anti-tumor effect [16]. A recent report by Rivadeneira et al*.* demonstrated that OVs engineered to express the adipokine leptin boosted T cell metabolic function in the TME, and thereby allowed a superior antitumor response [15]. Dendritic cells played important role in oncolytic virotherapy. Cytopathogenic infection of neoplastic cells releases the proteome and exposes pathogen- and damage-associated molecular patterns. At the same time, sublethal infection of antigen-presenting cells, such as dendritic cells and macrophages, yields potent, sustained type I interferon-dominant activation in an immunosuppressed microenvironment and promotes the development of tumor antigen-specific T cell responses in vitro and antitumor immunity in vivo [111]. The recombinant poliovirus/rhinovirus chimera oncolytic virus PVSRIPO’s immune adjuvancy stimulates canonical innate anti-pathogen inflammatory responses within the TME that culminate in dendritic cell and T cell infiltration. The findings provide mechanistic evidence that PVSRIPO functions as a potent intratumor immune adjuvant and generates tumor antigen-specific cytotoxic T lymphocyte responses [111]. T-Vec results in a rapid eradication of malignant cells and leads to interferon pathway activation and early influx of natural killer cells, monocytes, and dendritic cells. These events are followed by enrichment in cytotoxic T cells and a decrease of regulatory T cells in injected and noninjected lesions [112]. High-grade serous ovarian cancers (HGSOCs) exhibit limited response to immune checkpoint blockade. In a new study in *Cancer Cell*, Duraiswamy et al*.* highlighted that intratumoral CD28 co-stimulation by myeloid-antigen-presenting cells as a key mechanism was required for activation of programmed cell death receptor 1 (PD-1)^+^ tumor-infiltrating T lymphocytes during PD-1 blockade in HGSOC [113, 114].

The destructive genes, e.g. pro-apoptotic and toxin genes, have been engineered with OVs successfully. For example, arming OVs with a secretable and self-multimerizing apoptosis inducer is a approachable strategy to enhance the potency of OVT. Loya et al*.* armed HSV with a secreted form of an Her2 single chain antibody linked to the Fas ligand extracellular domain (Her2-COL-sFasL), which improved the bystander effect of OVT effectively [17]. Arming human MSCs with oHSV and its pro-apoptotic variant, oHSV-TRAIL, proved to be efficient in treatment for malignant glioblastoma multiforme [115]. Therapy of experimentally induced lung melanoma in mice with IL-15-carrying myxomavirus delivered by MSCs led to marked regression of lesions and with increased animal survival, suggesting that it allowed for safe and effective delivery of OVs to pulmonary melanoma lesions triggering immune responses [83]. HSV1716 administration led to marked tumor shrinkage in primary mammary tumors and a decrease in metastases by reprograming tumor-associated macrophage to a less immunosuppressive phenotype. This was associated with a significant increase in the recruitment/activation of cytotoxic T cells [116]. A pro-apoptotic gene *p53* has been engineered in Ads to treat hepatocellular carcinoma (HCC) and could prolong the survival time of the patients [117]. Dual silencing of *Bcl-2* and *Survivin* with oHSV-1 was also a promising tool for improving the antitumor efficacy [118]. A toxin gene, staphylococcal enterotoxin A, is also a potential useful anti-tumor agent in arming Ads [119]. A virulence factor, helicobacter pylori neutrophil-activating protein (HP-NAP), can mediate antitumor effects by recruiting neutrophils and inducing Th1-type differentiation in the TME. Thus, Ads armed with *HP-NAP* gene provoked antitumor immune response and enhanced the therapeutic effect against neuroendocrine tumors [120]. The study demonstrated that the cancer-associated fibroblasts (CAFs) induced high levels of fibroblast growth factor 2 (FGF2), which enhanced the susceptibility of the cancer cells to OV infection and improved therapeutic efficacy [121]. Telomelysin, a telomerase-specific replication-competent Ads with hTERT promoter, has been proven to have a strong antitumor effect on a variety of cancers and applied in combination treatment for head and neck squamous cell carcinoma [122]. The control of exogenous gene expression can also improve OVT. Jochen Stritzker et al*.* has characterized a doxycycline-inducible promoter system in oVVs, which was proven to be beneficial to OVT [123]. Therefore, determination of the structure and characteristics of various viruses and tumor cells will be greatly beneficial for the development of efficient OVT.

Overall, in addition to edit the viruses and exogenous genes, to excavate the reasonable combinatorial modalities are regarded as an excellent strategy to improve efficiency, especially ICIs [124–127] (Tables 3 and 4). For example, T-VEC with ipilimumab (a CTLA-4 inhibitor) had a tolerable safety profile, and the combination appeared to have greater efficacy than either T-VEC or ipilimumab monotherapy [30, 35]. The combination of intratumoral G47Δ and systemic anti-CTLA-4 antibody was shown to recruit effector T cells into the tumor efficiently while decreasing regulatory T cells [128]. Viral replication and the creation of new T-cell clones have been detected during treatment with reovirus pelareorep combined with a PD-1 inhibitor pembrolizumab [129]. While anti-PD-1 antibody monotherapy moderately improved tumor survival, when co-administered with oncolytic Zika virus (ZIKV), survival extended [27].

**Table 4:** The clinic trials of OVs

Virus name	Oncolytic virus (short name)	Viral gene modification	Non-viral gene addition	Human Phases, (N)	Tumor type	Virus administration route, dose and times	Combination therapy	Safety	Efficacy, (n/N, CR, PR, SD, NR; Survival)	Novelty, advantages	Comments, disadvantages	Refs
HSV-1	T-VEC	ICP34.5 ICP47 deletion	GM-CSF	Phase Ib/II; 19	Melanoma	IT, week 1, 10^6^ pfus/mL; week 4 and every 2 wks, 10^8^ pfu /mL	Ipilimumab, IV, 3 mg/kg/3 wks 4x	Nausealipase amylase	18-month PF-SD, 50%; 18-month OS, 67%	A tolerable safety profile, and greater efficacy	AntigenspecificT cell not sure	[35]
HSV-1	Talimogene laherparepvec (T-VEC)	ICP34.5 ICP47 Deletion	GM-CSF	phase II,(198)	Melanoma, unresectable stages IIIB to IV	Wk 1, ≤ 4 mL × 10^6^ pfu/mL; after 3 wks, ≤ 4 mL, 10^8^ pfu/mL/2 wks	Ipilimumab, 3 mg/kg/3 wks 4x	Fatigue chills, diarrhea	Greater antitumor activity versus ipilimumab	This was the first randomized trial of an OV plus checkpoint inhibitor	Phase II only	[34]
HSV-1	T-VEC	ICP34.5 ICP47 deletion	GM-CSF	Phase III, 436	Melanoma, unresected stages IIIB-IV	IT; 2.8 ml, 2 times			Tumor decrease ≥ 50% in 64% injected, 15–34% uninjected	Response in injected and uninjected lesions	Mechanisms unclear	[99]
HSV-1	T-VEC	ICP34.5 ICP47 deletion	GM-CSF	41 patients	Melanoma unresected, stage IIIB-IVM1c	IT, 4 ml × 10^6^ pfu/ml at day 1, 4 ml × 10^8^ PFU/ml/2wks 21 days later		Vomiting, abdominal pain, chills, hyperhidrosis, pyrexia	ECOG performance of 0 (68%) or 1 (32%). Median treatment 13.1 wks (3.0–41.1)	A comparable safety profile	Study endpoints limited	[25]
HSV-1	T-VEC	ICP34.5 ICP47 del	GM-CSF	Phase 1, 27	Melanoma IIIB–IV	IT, 10^6^ pfu/mL HSV-naïve, 10^8^ 3 wks later, every 2 weeks until DP/DLT	PD-1 inhibitor		Most only mild symptoms, fever and chills	Higher response rate than OPTiM, response associated with lesion size	Limited sample size	[134]
HSV-1	T-VEC	ICP34·5 ICP47 del	GM-CSF	Phase 2, 60	Melanoma advanced	IT, 10^6^ PFU/mL, 10^8^ 21 d later and every 14 d thereafter		Chills, flu-like symptoms		Extensive on the intratumoral distribution and transmissibility		[131]
HSV-1	HSV1716	ICP34.5 (RL1), mutation		Phase I, 9	Extracranial cancers, Pediatric cancer	IT, 10^5^–10^7^ pfu 1–4 doses		Fever, chills, cytopenia systemic viremia		Tolerable safety	Virus persistence not clear	[178]
HSV-1	HSV1716	ICP34.5 Del	TK	Phase I/IIa,13	MPM	Intrapleural, 10^7^ iu, 1, 2 or 4 times/wk	Cisplatin	Worst CTCAE, grade 1 for 46%; grade 2 for 46; grade 3, 8%	SD, 2/each, PD, 1–4	Future immune checkpoint inhibitor combination	Patients limited	[133]
HSV-1	HF10			Phase I, 12	Pancreatic cancer, unresectable locally advanced	IT, EUS 1/4wks, -4 × unless DLT appears	Erlotinib gemcitabine		3 PR, 4 SD, 2 PD	Safe treatment		[179]
HSV-1	Seprehvir HSV171	ICP34.5 /RL1 mutation		Phase I, 9	Solid tumors, non-CNS	IT, 5 × 10^4^ -2 × 10^6^ iu/kg or IV 2.5 × 10^5^ -2 × 10^7^ iu/kg		1, grade 3 hypotension, flu-like symptoms, 1, mild bleeding	Well tolerated, promising anti-cancer efficacy	First IV Seprehvir in Young Patients	Not clear IT or IV better	[132]
HSV-1	OrienX010		GM-CSF	Phase I, 12	unresectable stage IIIC–IV melanoma	10 mL of 8 × 10^7^pfu/mL OrienX010 IT injections every 2 weeks		Only one patient experienced a grade ≥ 3 adverse event and no dose limiting toxicities were observed	The median progression-free survival was 2.9 months and overall survival was 19.2 months	safe and well tolerated with a positive trend of antitumor effects	A larger clinical trial is warranted to validate the results of this study	[180]
HSV-1	G47Δ	Deletion the α47 gene and overlapping US11 promoter, γ34.5 gene and ICP6 gene		Phase II, 19	residual or recurrent glioblastoma	IT, 1 × 10^9^ p.f.u. per dose in 1 ml and repeatedly for up to six doses	radiation therapy, temozolomide, bevacizumab	fever (17 of 19) followed by vomiting, nausea, lymphocy topenia and leukopenia	The 1-yr survival rate of 84.2% and the median OS and PFS of 20.2 months and 4.7 months, respectively	the first oncolytic virus drug in Japan	The study population was rather small	[135]
HSV-1	G47Δ	Deletion the α47 gene and overlapping US11 promoter, γ34.5 gene and ICP6 gene		Phase I/II, 13	Progressive glioblastoma	IT, 3 × 10^8^ pfu (low dose) or 1 × 10^9^ pfu (set dose), twice to identical coordinates within 5–14 days	radiation and temozolomide therapies	fever, headache and vomiting	Median overall survival was 7.3 (95%CI 6.2–15.2) months and the 1-year survival rate was 38.5%	tumor cell destruction via viral replication and lymphocyteinfiltration towards tumor cells		[136]
Ad	Enadenotucirev	E2B substitution Ad3 to Ad11, E3 del, 25 bp del in E4orf4		Phase I, 17	CRC, NSCLC, UCC, RCC	IT (CRC) ≤ 3 × 10^11^ vp on d1; IV, 3 doses 1 × 10^12^ vp on d1/3/5		Asthenia, neutropenia, chills, pyrexia	High local CD8^+^ cell infiltration in 80% tumors	Safety, targeting, kinetic, immunology		[142]
Ad	Enadenotucirev	E2B Ad3 for Ad11; E3 del, E4orf4 25 bp del		Phase I, 61	Colorectal cancer	IV, 1 × 10^10^ vp/5 min on days 1, 3, and 5		Pyrexia, chills, hypoxia, lymphopen-ia, neutropenia		MDT tedermined	only limited information antitumor activity	[67]
Ad	Enadenotucirev	E2B Ad3 for Ad11; E3 del, E4orf4 25 bp del		Phase I, 30	Colorectal cancer, advanced	IV, 1–3 × 10^12^ vp, 3 × , wks 1–2, prior to chemoradiotherapy	Chemoradiation	No more than 30% probability of a DLT	Very high selectivity for colorectal cells	Administered systemically	Statistical support	[143]
Ad	DNX-2401 (Delta-24-RGD; tasadenoturev)	E1A 24-bp del	RGD-motif into the fiber H-loop	Phase I, 37	Glioma, malignant recurrent	Stereotactic IT via implanted catheter (10^7^ -3 × 10^10^ vp)		No dose-limiting toxicities observed	OS, 3y, 20%	Direct oncolytic effect + antitumor immune response		[69]
Ad	DNX-2401	E1A 24-bp del	RGD-motif into the fiber H-loop	Phase I, 12	Glioma, diffuse intrinsic pontine	Cerebellar peduncle biopsy, IT 5 × 10^10^	Radiotherapy and chemotherapy	Grade III-IV, secondary to dose dense temozolomide				[68]
Ad	DNX-2401	E1A 24-bp del	RGD-motif into the fiber H-loop	Phase I, 12	Diffuse Intrinsic Pontine Gliomas	Cerebellar peduncle, 1 × 10^10^ or 5 × 10^10^ viral particles of DNX-2401,	radiotherapy	headache, nausea, vomiting, and fatigue; Hemiparesis and tetraparesis developed in 1 patient each	a reduction in tumor size, was reported in 9 patients, a partial response in 3 patients, and stable disease in 8 patients	Resulted in changes in T-cell activity and a reduction in or stabilization of tumor size in some patients	associated with adverse events	[181]
Ad	DNX-2401	E1A 24-bp del	RGD-motif into the fiber H-loop	Phase I, 20	recurrentglioblastoma	locally delivered by convection enhanced delivery, 10^7^ to 1 × 10^11^ viral particles		In 14 patients, 17 serious AEs occurred, of which 8 were unrelated to the study treatment	Median PFS was 82 days, with a median OS of 129 days; One patient with complete regression and still alive after 8 years	The first to assess the local and locoregional responses upon infusion of an oncolytic virus into the tumor and surrounding brain by sequential sampling of brain interstitial fluid and cerebrospinal fluid		[182]
Ad	ICOVIR-5	E1A-Δ24 deletion	DM-1 insulator, E2F1 promoter, Kozak seq, RGD fiber	Phase I, 12	Melanoma	IV, 1a, 1 × 10^11^ vp, 2a, 3.3 × 10^11^, 3a, 10^12^, 4a, 3.3 × 10^12^, 5a, 10^13^ vp			Reached metastases but no tumor regression	the MTD determined	Necessary to arm the oAd	[139]
Ad	ICOVIR-5	E1A-Δ24 deletion,	DM-1 insulator, E2F-1 promoter, Kozak seq, RGD fiber	Phase I, 16	Solid tumors, relapsed or refractory	IV, weekly infusions 6 wks, 2 × 10^6^ cells/kg children, 0.5–1 × 10^6^ cells/kg adults, 2 × 10^4^ vp/cell	MSC	In pediatric patients, grade 1 fever headache; In adult patients, grade 1 fever asthenia	Two patients showed SD	Safe	Antiviral immune response may limit the effects	[82]
Ad	Aglatimagene besadenovec (AdV-tk)		*TK* gene	Phase 1, 8	Glioma, malignant;recurrent ependymoma	IT, 10^11^ and 3 × 10^11^ vp	Valacyclovir; radiation therapy, temozolomide	Grade 1–2 fever, fatigue, and nausea/vomiting	3, survived 24 m; 2 PFD at 37.3 and 47.7 m	The first study of GMCI in pediatric CNS tumors	The study population was small	[141]
Ad	VCN-01	E1A-Δ24 deletion	E2F1 promoter	Phase I, 2	Retinoblastoma	Intravitreous inject., Twice 14d interval, 2 × 10^9–10^ vp/eye, 1/10-100MFD		No systemic AV and viral genomes in blood	Have anti-tumor activity	provide a tumor- selective treatment option	Local vitreous inflammation	[140]
Ad	CG0070	E2F-1 promoter/E1A	GM-CSF	Phase II trial, 35	NMIBC, high-grade	2 h DDM pretreatment, 10^12^ Vp/100 mL saline/45–50 min/wk via a 100% silicone 3-way catheter, intravesical 6x		Bladder spasms, hematuria, dysuria, urgency, dysuria, hypotension	47% CR 50% CR for CIS	Tolerable safety, replication, GM-CSF expression	Relatively small sample size and short follow-up	[93]
Ad	NSC.CRAd-S-pk7		Survivin promotor, poly-L-lysine (pk7)	Phase I, 12	Glioma	6·25 × 10^10^ vp/5 × 10^7^ NSCs, 1·25 × 10^11^ vp/10^8^, or 1·87 × 10^11^ vp/1.5 × 10^8^	NSCs	Grade 3 viral meningitis due to the inadvertent injection	PFS, 9·1 m; OS, 18·4 m	1·875 × 10^11^/1·50 × 10^8^ NSCs for phase 2 trial	Replication conditional upon surviving	[65]
VV	GL-ONC1		Ruc-GFP, β-glucuronidase, and β-galactosidase	Phase I, 19	Head/neck carcinoma, locoregionally advanced unresected nonmetastatic	IV. Day 3, cohort 1, 3 × 10^8^ pfu; cohort 2, 1 × 10^9^ pfu; cohort 3, 3 × 10^9^ pfu; cohort 4, 3 × 10^9^ pfu, on d3, 8; 4 doses in cohort 5, d3, 8, 15, 22	Cisplatin Radiotherapy	Grade 1–2 rigors, fever, fatigue, and rash. Grade 3 hypotension, mucositis, nausea, vomiting	1y (2y) PFS and OS were 74.4% (64.1%) and 84.6% (69.2%), respectively	This is the first clinical trial for head and neck cancer	Limited benefit of repeated administrations of virus	[148]
VV	GL-ONC1		Ruc-GFP, β-glucuronidase, β-galactosidase	Phase I, 9	PC or PM	IP, 10^7^–10^9^ pfu/4 wks, 4x, dose escalation		Transient flu-like symptoms, abdominal pain		First-in-man intraperitoneal (IP)	Infection limited to treatment cycle 1	[149]
VV	TG4023 (MVA-FCU1)		Yeast FCU1	Phase I, 16	Liver tumors	IT, 107, 108, or 4.108 pfu, a DLT-driven 3 + 3 dose-escalating	5-FC	Pyrexia, asthenia, vomiting, decreased appetite	5FU = 1.9 ± 2.6 ng/ml/sera, 56 ± 30 ng/g/tumor. FCU1 found	Safe, MTD = 4 × 108 pfu, high 5-FU in tumors		[147]
VV	ACAM2000		*tk*-positive oVV	Phase I, 26	AML, stage III or IV	Incubated VV with SVF for 15–60 min	Adipose stromal vascular fraction cells	Self-limiting skin rashes	Well tolerated	First-in-human study		[80]
VV	Olvi-Vec			Phase 1b,12	PRROC	Intraperitoneal, 3 × 10^9^ (n = 6), 1 × 10^10^ (n = 5), and 2.5 × 1010 (n = 1) PFU/day on two consecutive days		There were no Grade 4 TRAEs, no dose relationship to TRAEs, and no deaths attributed to Olvi-Vec	Median PFS was 15.7 weeks	safety, clinical activities, and immune activation		[183]
VV	JX‑594		GM-CSF	Phase II,20	Advancedsoft‑tissue sarcoma	Intra-venously at the dose 1.109 every 2 weeks for the first 3 injections and then every 3 weeks	Cyclophosphamide	The two most frequent toxicities were grade 1 fatigue and fever and grade 2 fatigue and grade 2 lymphopenia in arms 1 and 2, respectively	One patient out 4 assessable for efficacy was progression-free at 6 months in arm 2	Cyclophosphamide and JX-594 could have a synergistic antitumor, and immuno-stimulating activity	The first stage of the Simon’s design was not satisfied	[95]
MV	MV-NIS		NIS	Phase I, 32	MM	Infusion in 250 ml saline/60 min	Cyclophosphamide	Neutropenia, leukocyte down, thrombocytopenia, anemia	CR (1); serum FLCs drops; MV-NIS replicated	Safe and novel approach for relapsed and refractory disease	Small sample size	[150]
MV (Edmonston strain)	MV-NIS		NIS	Phase I, 32	Melanoma metastatic	IV, 10^6–11^ iu/patient		MTD was not reached	Increased T-cell responses against MAGE-C1 MAGE-A3	Future combination with immune checkpoint inhibitor		[92]
MV	MV			phase I, 10	GBM	IT, on day 1 and 5 via a catheter				Prediction algorithm for oncolytic treatment	Validation limited	[184]
Parvovirus	ParvOryx			Phase II, 7	Pancreatic cancer, metastatic	IV, 40% dose in 4 days, 60% IV, 1, hepatic m	Gemcitabine, nab-paclitaxel		Pronounced anti-tumor effects	Further crucial information		[153]
Parvovirus	H-1 Parvovirus,ParvOryx01			Phase I/IIa, (18)	GBM, recurrent	Escalating dose, IT or IV injection at 1 and 9 days			Median survival extended	Safety, tolerability, virus pharmacokinetics, shedding, MTD	Necrosis induction needs further study	[152]
Reovirus (type 3 dearing)	Pelareorep (REOLYSIN ®)			Phase II,14	Melanoma, metastatic	1 h intravenous infusion at a dose of 3 × 10^10^ TCID_50_	Paclitaxel carboplatin	Pyrexia, grade 3 febrile neutropenia (1)	SD = 85%, PFS and OS = 5.2 and 10.9 m, 1-year OS 43%	Safe and potentially efficacious		[126]
Reovirus	Pelareorep			Phase II,74	Breast cancer, metastatic	IV, 3 × 10^10^ TCID_50_/4 wks on days 1, 2, 8, 9, 15, and 16	Paclitaxel	fever fatigue diarrhoea chills nausea “flu-like”	PFS increase from 4 to 7.5 m in 67	The first randomized phase II trial	The trial did not demonstrate a benefit	[185]
Reovirus	Pelareorep			Phase I,11	PDAC	4.5 × 10^10^ TCID50 IV on days 1 + 2 after chemotherapy	Pembrolizumab, 2 mg/kg IV on day 8	Grade 3 or 4 TRAEs neutropenia/leukopenia /myalgias/fever/chills	PFS = 2 m OS = 3.1 m 1/2-year survival = 35%/23%	Not add significant toxicity, encouraging efficacy	Small sample size	[129]
Coxsackievirus	Coxsackievirus A21 (V937)			Phase II, 57	unresectable stage IIIC or IV melanoma	3 × 10^8^ TCID50 in a maximum 4.0-mL volume by intratumoral injection		No treatment-related grade ≥ 3 adverse events occurred	6-month PFS rate per irRECIST, was 38.6%	V937 was well tolerated	combination with immune checkpoint inhibitors are ongoing	[186]

## OVs in clinical trials

Although the pre-clinical trials so far have established the safety and efficacy of those approaches, the challenge now is to achieve safety and efficacy in clinics. Many promising OVs, such as oHSVs, oAds, and oVVs, have been applied in clinic trials successfully (Table 4).

T-VEC, a recombinant oHSV, which is administered by direct I.T. injection to patients with metastatic malignant melanoma led to lesion regressions of [30, 34, 35, 51, 96, 99, 130]. As an example, the biodistribution, shedding, and potential transmission of T-VEC have been systematically evaluated during and after completion of therapy in adults with advanced melanoma [131]. The data demonstrated that T-VEC improved longer-term efficacy versus GM-CSF and maintained well tolerated. The final planned OPTiM analysis suggested that the median OS was 23.3 months (95% confidence interval [CI] 19.5–29.6) and 18.9 months (95% CI 16.0–23.7) in the T-VEC and GM-CSF arms, respectively [130]. A phase II study evaluated patients with unresectable stage IIIB-IVM1c malignant melanoma who received T-VEC plus ipilimumab or ipilimumab alone. The results showed that 39% (n = 38/98) in the combination arm and 18% (n = 18/100) in the ipilimumab arm had an objective response. Eight responders (combination, n = 7 [18.4%]; ipilimumab, n = 1 [5.6%]) had pseudo-progression; most occurred by week 12 and were caused by an increase in existing lesions [30]. In addition, to determine the safety of administering HSV1716 (Seprehvir) systemically, Streby et al*.* conducted the phase I trial of intravenous (I.V.) injection in young patients with relapsed or refractory extra-cranial solid cancers [132]. They did not observe any dose-limiting toxicities. All five HSV-1 seronegative patients seroconverted by day 28. Four out of nine patients had detectable HSV-1 genomes in peripheral blood on day + 4, which is consistent with de novo virus replication. A phase I/IIa trial of intrapleural administration of HSV1716 with malignant pleural mesothelioma patients demonstrated that viral replication/persistence in pleural fluid in seven of the twelve patients. Induction of Th1 cytokine responses to HSV1716 treatment was achieved in eight patients and four patients developed novel anti-tumor IgG [133]. However, it is also suggested that the efficacy of T-VEC therapy in patients with in-transit melanoma metastasis diminished with increasing lesion size [134]. Of 27 patients, an objective response was observed in 11 (40.7%), including one patient with partial response (3.7%) and 10 with complete response (37.0%). Logistic regression demonstrated each millimeter increase in maximum lesion diameter predicted decreased ORR (odds ratio [OR] 0.866, 95% CI 0.753–0.995; p = 0.04) [134]. Todo et al. have been reported the results of a phase I/II trial using triple-mutated oHSV-1 G47Δ in Japanese patients with recurrent or progressive glioblastoma [135, 136]. G47Δ caused immediate infiltration of lymphocytes that seemingly directed towards tumor cells, which was reflected on image studies with features characteristic to G47Δ therapy. Long-term survival (> 46 months) was observed in 3 of 13 patients, which may be due to the delayed effect of G47Δ via antitumor immunity [136].

Since that first approve of the human p53 adenovirus (Gendicine), a steady stream of new oAds entering the clinical arena [137, 138]. Clinical studies demonstrated that DNX-2401 is safe and tolerable after injection into the cerebellar peduncle in pediatric patients with diffuse intrinsic pontine gliomas and can induce a direct oncolytic effect followed by an antitumor immune response [68]. ICOVIR5 was derived from the oAd DNX-2401. The clinical results in 12 patients treated with a single dose up to 1 × 10^13^ viral particles showed that ICOVIR5 was able to reach melanoma metastatic lesions after infusion but failed to induce tumor regressions [139]. The homing capacity of MSCs to tumors makes them excellent carriers of anticancer therapeutics [40, 44]. Autologous MSCs may allow an increasing amount of ICOVIR5 by repeated administration, avoiding or minimizing emergent toxicities [82]. Evidence have been reported that MSCs successfully delivered an oAd CRAd-S-pK7 with fiber modification of seven lysine residues to diffuse intrinsic pontine glioma [71]. Similarly, it is shown to protect CRAd-S-pK7 from neutralizing antibodies within patient ascites fluid and to enhance delivery of CRAd-S-pK7 by NSCs for treatment of metastatic ovarian cancer [84]. Recently, the safety and feasibility of NSC-CRAd-S-pk7 in patients with newly diagnosed high-grade glioma have been examined, and the results showed that the median progression-free survival was 91 months (95% CI 85-not reached) and median OS was 184 months [65]. In addition, Pascual-Pasto et al*.* confirmed that the oAd VCN-01 provided targeted therapeutic activity against even chemo- resistant retinoblastoma. The phase I data in patients showed the feasibility of the administration of intravitreous VCN-01 and resulted in antitumor activity in retinoblastoma vitreous seeds and evidence of viral replication markers in tumor cells [140]. In another phase I study of gene-mediated cytotoxic immunotherapy using aglatimagene besadenovec (AdV-tk), an adenoviral vector expressing the *HSV-tk* gene, followed by valacyclovir, 3 patients in a dose of level 2 (3 × 10^11^ vp) survived more than 24 months after treatment, and 2 remain alive without progression at 37.3 and 47.7 months after AdV-tk injection [141]. Enadenotucirev is a tumor selective oAd, which can be administrated intravenously in patients undergoing primary tumor resection [142]. Additionally, the EVOLVE (Evaluating Oncolytic Vaccine Efficacy) study of the enadenotucirev, administered intravenously to patients with epithelial solid tumors, showed that enadenotucirev monotherapy can be administered in a single cycle or repeated cycles with manageable tolerability [67]. Recent clinic trial confirmed that enadenotucirev is a radiosensitizer in chemoradiation therapy of locally advanced rectal cancers [143]. Intravenously dosed enadenotucirev plus paclitaxel demonstrated manageable tolerability and increased tumor immune-cell infiltration in phase 1 studies in platinum-resistant ovarian cancer [144].

An oVV, Pexa-Vec (pexastimogene devacirepvec, JX-594), engineered to express GM-CSF, was administered IT and IV to patients with HCC and colorectal cancer, respectively [94, 145, 146]. No dose-limiting toxicity (DLT) was reported, and the maximum tolerated dose was not reached in phase Ib trial of biweekly IV of Pexa-Vec. Moreover, the most common adverse events were grade 1/2 flu-like symptoms, generally lasting less than 24 h [146]. TG4023 is a modified vaccinia virus Ankara (MVA), the first-in-human study demonstrated that IT injections of TG4023 were feasible and well tolerated, and the maximum tolerated dose (MTD) was defined as 4 × 10^8^ p.f.u. [147]. The safety of oVV GL-ONC1 have been determined when delivered intravenously with chemoradiotherapy to patients with primary, nonmetastatic head and neck cancer [148]. Moreover, the study showed that GL-ONC1 was well tolerated when administered into the peritoneal cavity of patients with advanced stage peritoneal carcinomatosis. Importantly, in 8 of 9 studied patients, effective peritoneal infections, in-patient replication of GL-ONC1, and subsequent oncolysis were detected [149]. ACAM2000, a TK-positive strain of oVV, is the current smallpox vaccine in the US. The phase I clinical trial confirmed that ACAM2000/SVF can safely be administered in patients with advanced metastatic solid tumors or advanced AML [80].

In addition to the above described oHSVs, oAds, and oVVs, an oMV engineered to express the human thyroidal natrium iodine symporter (MV-NIS) monitors localization of viral gene expression and successfully used in clinical trials against multiple myelomas and ovarian cancers [92, 150, 151]. Packiriswamy et al*.* conformed that MV-NIS treatment significantly (P < 0.05) increased cytotoxic T-lymphocyte responses against TAAs in patients with MM [92]. An oncolytic parvovirus ParvOryx containing native parvovirus H-1 (H-1PV) have been shown to be a promising candidate for treatment of patients with recurrent glioblastoma and metastatic, inoperable pancreatic cancers [152, 153]. Pelareorep, an oncolytic reovirus, in combination with chemotherapy and pembrolizumab in patients with advanced, pre-treated pancreatic ductal adenocarcinoma (PDAC) was well-tolerated and showed prolonged efficacy in 3 of 11 patients (27.3%) [129].

Despite the confirmed safety and antitumor efficacy of OVs, additional challenges have been gained from the ongoing and completed clinical trials. A first insight is that the predictive values including safety and efficacy profile are limited by the relatively small sample size of patients and short follow-up. A second awareness is that the antigenic specificity of the T cell response to these OVs has not been determined, and whether the treatment expands the appearance of new antigen specific T cell lineages; further research is required to monitor/determine any relationship between virus persistence and the TME. Third, the role of adaptive immunity in restricting the benefits of repeated administrations of viruses is unknown. In addition, it is not clear which administrations of OVs is better, injecting the tumor intratumorally, intravenously, or orally, which may vary depending on the individual tumors, viruses, patients, and combination therapy regimen.

Significantly, Gendicine is the first OV approved for clinical OVT in the world in 2003 [137, 138], which was approved for head and neck carcinoma by China FDA and T-VEC is the second OV approved for clinical OVT in the world in 2015 [154], which was approved for melanoma by the US FDA. Many promising OVT clinical trials are under way but there is still a long way off to improve their safety and efficiency.

## Conclusions

OVT is an amazingly versatile and malleable class of cancer therapy, which has the unique advantages when compared with that in conventional therapies. OVs can attack tumor cells selectively, and then trigger the cell death by multiple approaches, including direct oncolytic effects, targeting blood vessel endothelial cells, delivery of the therapeutic genes within tumors, synergistic effects with traditional and immunotherapies, resulting in systemic anticancer effects. The toxicity of OVs has been self-limiting flu-like illness and fever etc. Until now, OVT has become a realistic therapeutic candidate, and has been evaluated for safety by both localized and systemic administration in clinics. From the previous studies, we conclude that the status of OVs potencies including: (i) induces systemic tumor-specific immunity, (ii) synergistic effects with other therapies, (iii) different tumor sites and patients showed varying response to different viruses, (iv) neutralizing antibody is not a barrier to successful therapy; and (v) anti-tumor T cell (BiTAs, checkpoint inhibitory T-cell-activators/CiTAs) or NK cell (trispecific killer activators, TriKAs) responses augment antitumor efficacy by OVTs.

Oral, I.V., I.T., intrapleural, intraperitoneal (IP), aerosol and limb injections are the common delivery routes for OVs. However, these methods still have their own disadvantages. To be specific, oral administration is most convenient and most unavailable. I.V. and I.T. injections are easy to be neutralized in blood stream of patients. Besides, not all patients can be adapted to I.T. injection. Intrapleural injection should be utilized by using an indwelling intrapleural catheter. To avoid uncontrolled adverse events and long-term complications of OVs, the patients need to orchestrate the appropriate time and delivery routes in clinics.

We believe OVT has a bright future and requires continue efforts working for its safety and efficiency. It is wise to explore the key factors affecting the efficacy of OVs from three aspects: virus, tumor and patient. This include reconstructing the viruses for better efficiency with more safety, utilizing intrinsic tumor-associated genes for target specificity, invoking immune responses from host for enhanced tumoricidal effect. To further avoid host immunity to viruses or enhance tumor specific immunity induced by OVs in the future, the potential novel investigations should be focusing on the following aspects: (i) sequential harness of two different OVs, (ii) choreographed combination of OVs and antibody therapies (anti-PD-1/PDL-1, anti-CTLA-4), or cell therapies (adoptive cell transfer therapy, DC, Car-T), and (iii) improve the efficacy of administration and delivery by excellent cell carriers (MSCs, NSCs, etc.).

## Data Availability

Not applicable.

## Abbreviations

PDAC: Pancreatic ductal adenocarcinoma
PKR: Double-stranded RNA-dependent protein kinase
CPEBs: Cytoplasmic polyadenylation element-binding proteins
ANTXR1: Anthrax toxin receptor 1
ICOS: Inducible co-stimulator
TIL: Tumor infiltrated leukocyte
TAA: Tumor associated antigen
scFv: Single-chain antibodies
CPEB: Cytoplasmic polyadenylation element-binding protein
HCC: Hepatocellular carcinoma
EGFR: Epidermal growth factor receptor
FR: Folate receptor
PSMA: Prostate membrane-specific antigen
HIF: Hypoxia-inducible factor
NSCLC: Non-small cell lung cancer
MOIs: Multiplicities of infection
NDV: Newcastle disease virus
SVV: Seneca Valley virus
TME: Tumor microenvironment
VSV: Vesicular stomatitis virus
ZIKV: Zika virus
Nabs: Neutralizing antibodies
RCA: Regulators of complement activity
TPMV: Tupaia paramyxovirus
CAFs: Cancer-associated fibroblasts
FGF2: Fibroblast growth factor 2
MSC: Human mesenchymal stem cells
sECM: Synthetic extracellular matrix
EnAd: Oncolytic group B adenovirus EnAdenotucirev
BiTA: Bispecific T-cell activator
DARPins: Designed ankyrin repeat proteins
CSC: Cancer stem cell
MSCs: Mesenchymal stem cells
NIS: Human thyroidal sodium-iodide symporter
RT3D: Reovirus serotype 3 Dearing
RUX: Ruxolitinib
MPNSTs: Malignant peripheral nerve sheath tumors
PARPi: Poly(ADP-ribose) polymerase inhibitors
HR: Homologous recombination
TGF-β: Transforming growth factor beta
LPS: Lipopolysaccharide
HP-NAP: Helicobacter pylori neutrophil-activating protein
EnAd: EnAdenotucirev
BAI1: Brain Angiogenesis Inhibitor 1
hTERT: Human telomerase reverse transcriptase
cBiTA: EGFR-targeting BiTA
G47Δ-mIL12: OHSV G47Δ expressing murine IL-12
ICOS: Inducible co-stimulator
NDV: Newcastle disease virus
NDV-ICOSL: NDV-expressing ICOS ligand
AE: Adverse events
PGE2: Prostaglandin E2
HPGD: Hydroxyprostaglandin dehydrogenase
VV: Vaccinia virus
SCLC: Small cell lung cancer
IP: Intraperitoneal
BiTA: Bispecific T-cell activator
UV-HSV-1: UV light-inactivated HSV-1
TRAIL: TNF-related apoptosis-inducing ligand
IT: Intratumoral
IV: Intravenous
oAd-MSCs: Oncolytic adenovirus dlE102
MM: Multiple myeloma
T-VEC: Talimogene laherparepvec
MTD: Maximum tolerated dose
PoC: Proof-of-concept
DLT: Dose-limiting toxicities
NMIBC: Non-muscle invasivebladder cancer
PC: Peritoneal carcinomatosis
PM: Peritoneal mesothelioma
IT: Intratumoral
pfu: Plaque-forming units
PPR: Progression prior to response
MPM: Malignant pleural mesothelioma
